# Molecular basis for CesT recognition of type III secretion effectors in enteropathogenic *Escherichia coli*

**DOI:** 10.1371/journal.ppat.1007224

**Published:** 2018-08-17

**Authors:** Dustin J. Little, Brian K. Coombes

**Affiliations:** Department of Biochemistry & Biomedical Sciences, Michael G. DeGroote Institute for Infectious Disease Research, McMaster University, Hamilton, Ontario, Canada; University of Virginia School of Medicine, UNITED STATES

## Abstract

Enteropathogenic *Escherichia coli* (EPEC) use a needle-like injection apparatus known as the type III secretion system (T3SS) to deliver protein effectors into host cells. Effector translocation is highly stratified in EPEC with the translocated intimin receptor (Tir) being the first effector delivered into the host. CesT is a multi-cargo chaperone that is required for the secretion of Tir and at least 9 other effectors. However, the structural and mechanistic basis for differential effector recognition by CesT remains unclear. Here, we delineated the minimal CesT-binding region on Tir to residues 35–77 and determined the 2.74 Å structure of CesT bound to an N-terminal fragment of Tir. Our structure revealed that the CesT-binding region in the N-terminus of Tir contains an additional conserved sequence, distinct from the known chaperone-binding β-motif, that we termed the CesT-extension motif because it extends the β-sheet core of CesT. This motif is also present in the C-terminus of Tir that we confirmed to be a unique second CesT-binding region. Point mutations that disrupt CesT-binding to the N- or C-terminus of Tir revealed that the newly identified carboxy-terminal CesT-binding region was required for efficient Tir translocation into HeLa cells and pedestal formation. Furthermore, the CesT-extension motif was identified in the N-terminal region of NleH1, NleH2, and EspZ, and mutations that disrupt this motif reduced translocation of these effectors, and in some cases, overall effector stability, thus validating the universality of this CesT-extension motif. The presence of two CesT-binding regions in Tir, along with the presence of the CesT-extension motif in other highly translocated effectors, may contribute to differential cargo recognition by CesT.

## Author summary

Enteropathogenic *Escherichia coli* injects effector proteins into host cells using a type III secretion system (T3SS). The translocated intimin receptor (Tir) is the first effector delivered into host cells and imparts efficient secretion of other effectors. However, the mechanism for Tir-dependent modulation of the T3SS is poorly understood. We provide evidence that the multi-cargo chaperone CesT binds to two regions in Tir at the N- and C-terminus through a specific recognition motif, and show that CesT binding to the Tir C-terminus is important for host translocation. Furthermore we show that the CesT-specific motif is conserved in a subset of highly translocated effectors. This study highlights the multi-faceted role that T3SS chaperones play in effector secretion dynamics.

## Introduction

Enteropathogenic and enterohemorrhagic *Escherichia coli* (EPEC and EHEC) cause acute gastroenteritis in humans and are a common source of outbreaks [1]. EPEC is a significant pathogen in the pediatric population, especially in areas with limited access to healthcare and clean water, whereas EHEC is a common food- or water-borne contaminant in industrialized nations [1]. EPEC and EHEC contain a genomic island called the locus of enterocyte effacement (LEE) that encodes a type III secretion system (T3SS) [2] necessary for the formation of attaching and effacing (A/E) lesions on epithelial cells [3]. The T3SS is a needle-like protein injectisome used by Gram-negative bacteria to deliver effector proteins into host cells directly from the bacterial cytosol [4], where they target specific host processes to allow for attachment, survival, and propagation of the bacteria [5, 6]. Enteric pathogens that use a T3SS for host attachment, infection, and/or colonization are significantly attenuated when lacking their encoded T3SS [7], identifying it as a key mediator of host-pathogen interactions.

Structural biology efforts have advanced our understanding of the assembly, structure, and function of the T3SS [8, 9]. The T3SS contains ~25 proteins assembled into distinct structures including, (*i*) an extracellular needle filament capped at the distal end by hydrophobic translocon proteins, (*ii*) a basal body comprised of inner and outer membrane-spanning rings, (*iii*) an ATPase-containing sorting platform complex at the cytoplasmic face of the basal body, and (*iv*) cytosolic chaperones that bind, protect, deliver, and control effector secretion [10]. Three classes of non-flagellar T3SS chaperones have been described. Class I chaperones bind translocated effectors, class II chaperones bind the hydrophobic translocators, and class III chaperones escort and prevent cytosolic polymerization of the extracellular needle filament [11, 12]. The class I chaperones are further subdivided into class IA and IB. Class IA chaperones are usually specific for one effector and are located adjacent to the gene that encodes the cognate effector [11, 13]. Class IA chaperones that bind multiple effectors have been reported, including EPEC CesT and *Salmonella* SrcA [14, 15], and are referred to as multi-cargo chaperones [16]. Class IB chaperones bind multiple effectors and are usually encoded within large operons that contain structural components of the T3SS instead of being adjacent to a specific effector gene [11, 13]. Class IB chaperones appear to be functionally interchangeable between species and recognize a specific sequence motif [17].

Multi-cargo chaperones play a significant role in T3SS-dependent infection biology as mutants lacking these proteins are attenuated in animal and plant models of infection [16]. CesT from EPEC and EHEC was originally thought to be a class 1A chaperone specific for the translocated intimin receptor (Tir) [18, 19]. However, it was later reclassified as a multi-cargo chaperone because it interacts with at least 9 other effectors [14, 20, 21], most of which require CesT for translocation into host cells [22, 23]. Recent work has indicated that the effector binding and secretion activities mediated by CesT can be functionally separated. For example, mutants in the C-terminal domain of CesT retain their ability to bind effector cargo, yet exhibit reduced effector secretion [24]. This C-terminal domain was also identified as a site for tyrosine phosphorylation in a phosphotyrosine-proteome study [25], in which tandem tyrosine phosphosites (Y152 and Y153) influenced NleA or global effector secretion, respectively [26]. Furthermore, host-cell contact has been proposed to liberate free CesT in the bacterial cytosol that can then bind and antagonize CsrA repression of the *nleA* 5’UTR [27]. This interaction is facilitated by the C-terminal domain of CesT [28], allowing for greater control over the timing and translocation efficiency of the NleA effector. Notwithstanding the requirement of CesT for effector secretion, Tir has been implicated in effector secretion hierarchy. Deletion of *tir* in the hyper-secreting Δ*sepD* strain of EPEC significantly reduced the level of at least 6 effectors in culture supernatants [21]. A similar but more modest effect was also seen for this subset of effectors translocated into host cells when only *tir* was deleted [23].

Given the primary importance of the Tir-CesT complex in orchestrating secondary effector secretion in *E*. *coli*, we initiated structural studies to characterize the Tir-CesT interaction and to delineate the role that this effector-chaperone pair plays in protein translocation. Here, we present the co-crystal structure of a C-terminal truncation of CesT in complex with an N-terminal fragment of Tir. This structure allowed us to define a CesT-extension motif, leading to the identification of a second CesT-binding region in the C-terminal domain of Tir, which we verified using biochemical and molecular assays. Furthermore, we identified the CesT-extension motif in the N-terminus of a subset of other effectors and demonstrated the function of this motif in effector translocation.

## Results

### Identification of the minimal CesT-binding region on Tir

**Fig 1:**
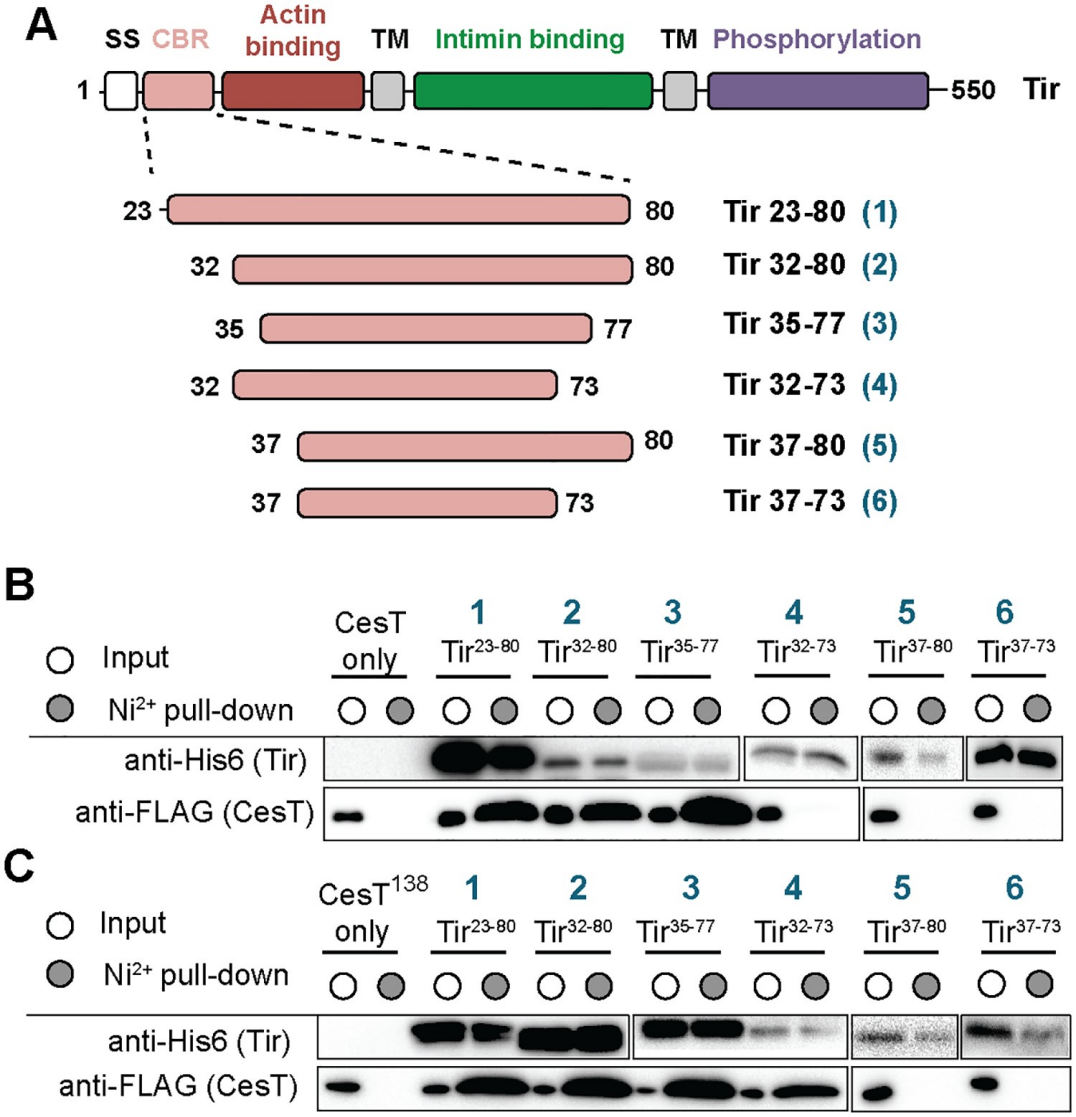
The minimal CesT-binding region is located between Tir residues 35–77. (A) Schematic representation of Tir and the N-terminal fragments used in the pull-down assays. SS, type III secretion signal; CBR, CesT-binding region; TM, transmembrane domain. His_6_-Tir peptides shown in (A) were co-expressed with (B) CesT-FLAG and (C) CesT^138^-FLAG. Cells were lysed, clarified by centrifugation, and the soluble cell lysates were applied to Ni^2+^ resin, extensively washed, and eluted. The soluble lysate (input, white circle) and Ni^2+^-pull-down (elution, grey circle) fractions were analyzed by SDS-PAGE and immunoblotting for the ability of the various Tir peptides to co-purify CesT or CesT^138^.

The first ~20 amino-terminal residues of *E*. *coli* T3SS effectors contain a T3SS-specific secretion signal that can be predicted bioinformatically [29]. Downstream of the T3SS secretion signal, but within the first ~100 residues, is an unspecified CesT-binding domain that has been identified in Tir, Map, and NleH [18, 20, 21]. Despite the fact that CesT binds to the N-terminus of these effectors, sequence alignments have not identified a consensus motif within this region. To determine the minimal recognition sequence of the CesT-binding region, various His_6_-tagged N-terminal Tir constructs were tested for their ability to co-purify CesT (Fig 1). Tir fragments containing residues 23–80, 32–80, and 35–77 co-purified CesT as seen by Ni^2+^-affinity pull-down and immunoblotting (Fig 1B and S1A Fig), whereas CesT alone was never pulled-down in the absence of Tir by the Ni^2+^-affinity resin, thus confirming specificity of our assay. When these Tir fragments were truncated further to residues 32–73, 37–80, and 37–73, they lost the ability to co-purify with CesT (Fig 1B). To determine the molecular basis of the Tir-CesT interaction, we carried out crystallization trials with the three Tir fragments that co-purified CesT (S1A Fig), however none of the complexes produced crystals. CesT contains a unique C-terminal extension that is not conserved among closely related chaperones, such as SrcA from *Salmonella* [15]. This C-terminal extension was shown to be important for effector secretion but was dispensable for effector binding [24]. We hypothesized that this C-terminal region of CesT was either disordered or heterogeneous from differential phosphorylation, possibly preventing favourable crystallization contacts. To address this, we truncated CesT at residue 138 (CesT^138^) and tested whether this variant could co-purify the same Tir peptides as full-length CesT. Tir peptides 23–80, 32–80, and 35–77 retained their ability to co-purify with CesT^138^ indicating that the C-terminus of CesT was not required for this interaction (Fig 1C and S1B Fig). However, the shorter Tir peptide 32–73 was now able to co-purify CesT^138^ (Fig 1C and S1B Fig), whereas the Tir peptides 37–80 and 37–73 were unable to co-purify CesT^138^. Gel filtration chromatography confirmed that both CesT and CesT^138^ existed in a dimeric configuration (S2 Fig), which is the functional unit of T3SS chaperones [30]. Taken together, these data suggest that the minimal CesT-binding region is located between residues 35–77 and that the C-terminus of CesT may interfere with binding of Tir residues 73–80.

### Tir^32-80^ interacts with CesT^138^ by extending the β-sheet core

**Fig 2:**
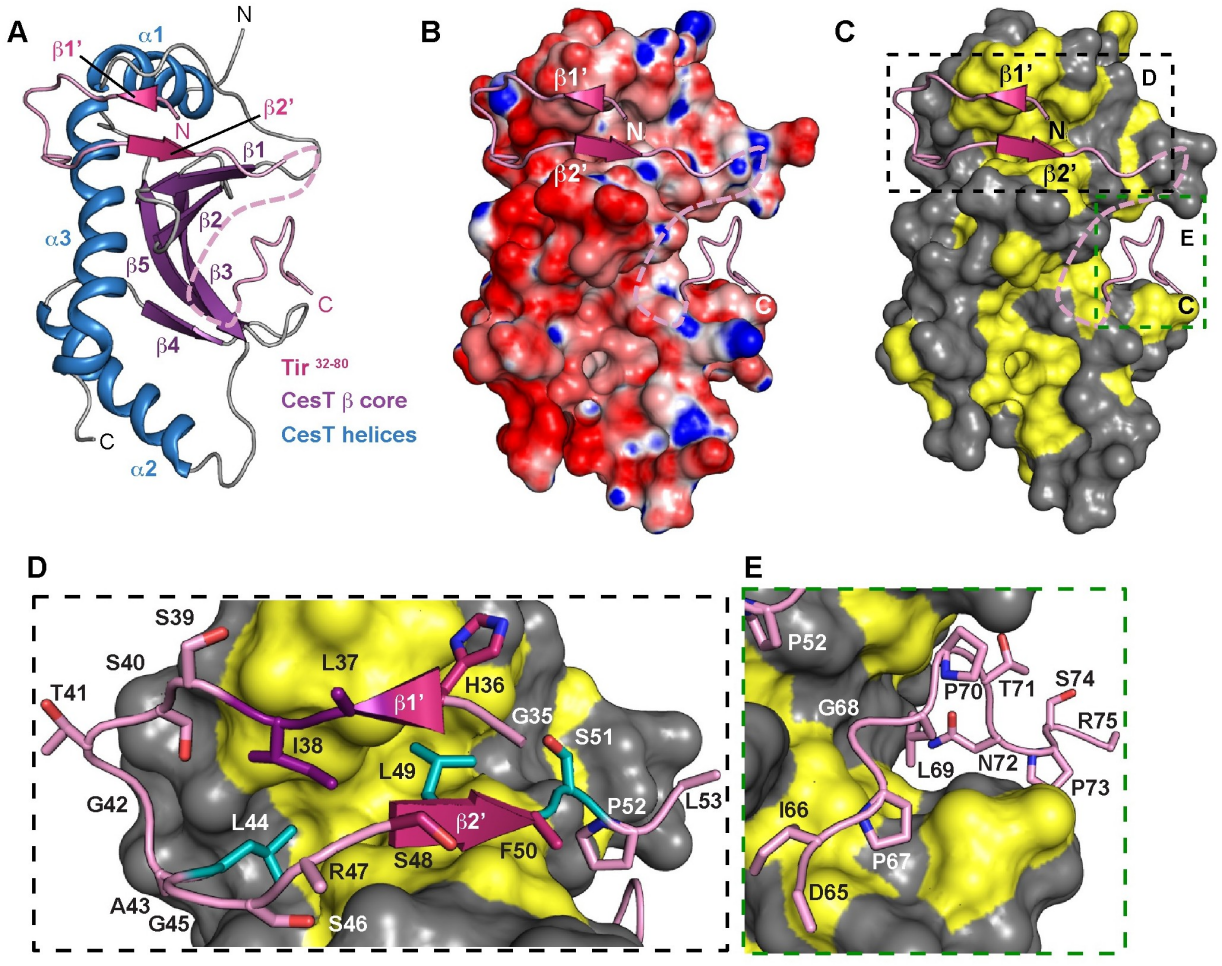
Structure of the Tir^32-80^-CesT^138^ complex. (A) Cartoon, (B) electrostatic surface potential, and (C) hydrophobic surface representation of CesT^138^ in complex with Tir^32-80^. CesT^138^ in panel A is coloured by secondary structural elements: β-strands (purple), α-helices (blue), and loops (grey); in panel B by quantitative electrostatics from red (-15 kT/e) to blue (+15 kT/e); and panel C by highlighting hydrophobic residues in yellow. Tir^32-80^ is shown in cartoon representation coloured pink, and residues 54–64 that could not be modeled are depicted as a dotted pink line. (D) and (E) show close-up views of the two Tir^32-80^ peptide binding regions from panel C. Tir residues are shown as pink sticks except for L37 and I38 which are purple, and L44, L49, and S51 from the β-motif coloured cyan.

The structural basis for how CesT binds and interacts with multiple T3SS effectors is not known. To determine the molecular determinants behind this interaction we conducted crystallization trials for all of the successful Tir-CesT co-purifications (S1 Fig). The Tir^32-80^-CesT^138^ complex produced crystals in the trigonal space-group *P*3_2_2 with one molecule of the complex in the asymmetric unit. Diffraction data were collected to 2.74 Å resolution and the structure was determined by molecular replacement (Table 1). Structural refinement produced a final model with good geometry and *R* factors (*R*_work_ and *R*_free_ of 20.8% and 25.5%, respectively) (Table 1). Residues 130–138 of CesT^138^, the N-terminal histidine tag, and residues 32–34, 54–64, and 76–80 of Tir were not included in the final model due to poor or absent electron density. Tir^32-80^ adopts minimal regular secondary structure that is limited to two small β-strands, β1’ and β2’ (Fig 2A). The Tir^32-80^ fragment binds CesT^138^ in two distinct locations and is separated by a break in the peptide chain likely due to residue mobility in the crystal. Tir residues 35–53 adopt a β-hairpin-like fold and extend the 5-stranded β-sheet core of CesT^138^, while also being pinched between α1 and orthogonally below by α3 of CesT^138^ (Fig 2A). The interaction between Tir^32-80^ β2’ and CesT^138^ occurs through a conserved 3-amino acid β-motif, adopting the consensus sequence of Φ-X_4_-Φ-x-Φ where x is any amino acid and Φ represents a hydrophobic residue. The β-motif was originally identified in the SipA-InvB complex [31, 32], but appears to be a conserved mode of binding present in all class I chaperone-effector complexes [33]. Slight differences have been observed in the β-motif, most notably that one to four residues can separate the first and second hydrophobic residues (ie. Φ-(X_1-4_)-Φ-x-Φ). Tir residues 65–75 are bound along the concave surface of the β-sheet core of CesT^138^ (Fig 2A). Despite CesT^138^ having a global acidic surface potential (Fig 2B), Tir^32-80^ binding is mediated through distinct hydrophobic-hydrophobic contacts (Fig 2C). Specifically, Tir residues I38 (purple), L44 (cyan), and L49 (cyan) anchor the β-hairpin-like peptide to CesT^138^ (Fig 2D); and L69 plus three additional proline residues make a second point of contact with the β-sheet core of CesT^138^ (Fig 2E).

**Table 1:** Summary of data collection and refinement statistics.

	Tir^32-80^-CesT^138^
**Data collection**	
Beamline	CLS 08B1-1
Wavelength (Å)	0.98
Space group	*P*3_2_2
Unit-cell parameters (Å)	*a* = 67.0, *b* = 67.0, *c* = 75.7
Resolution (Å)	58.00–2.74 (2.88–2.74) *
Total no. of reflections	36660
No. of unique reflections	5461
Redundancy	6.7 (6.5) *
Completeness (%)	99.7 (99.9) *
Average *I*/*σ*(*I*)	13.8 (2.2) *
*R*_merge_ (%)^a^	6.2 (80.1) *
**Refinement**	
*R*_work_^b^ / *R*_free_^c^	20.8 / 25.5
No. of protein atoms	1170
Average B-factors (Å^2^)^d^	130.7
RMS deviations	
Bond lengths (Å)	0.010
Bond angles (°)	1.35
Ramachandran plot^d^	
Total favoured (%)	93
Total allowed (%)	100
Coordinate error (Å)^e^	0.36
PDB code	5WEZ

*Values in parentheses correspond to the highest resolution shell

^a^*R*_merge_ = ∑∑ | *I* (k)—<*I*>| / ∑ *I* (k) where *I* (k) and <*I*> represent the diffraction intensity values of the individual measurements and the corresponding mean values. The summation is over all unique measurements.

^b^*R*_work_ = ∑ ||F_obs_|—k|F_calc_|| / |F_obs_| where F_obs_ and F_calc_ are the observed and calculated structure factors, respectively.

^*c*^*R*_free_ is the sum extended over a subset of reflections (5%) excluded from all stages of the refinement.

^d^As calculated using MolProbity [66].

^e^Maximum-Likelihood Based Coordinate Error, as determined by PHENIX [54]

### Structural alignment of the Tir^32-80^-CesT^138^ and CsrA-CesT complexes show marked differences in substrate binding

**Fig 3:**
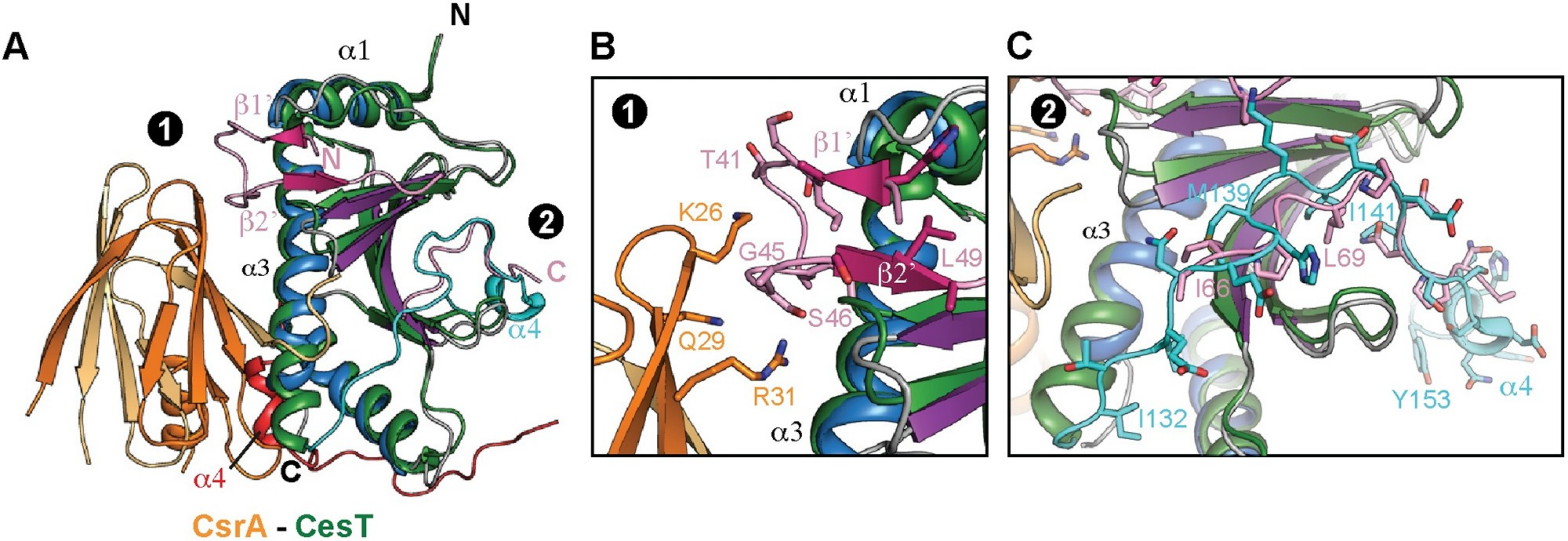
Structural comparison of the Tir^32-80^-CesT^138^ and CsrA-CesT complexes. Structural alignment of Tir^32-80^-CesT^138^ with the Re-CsrA-CesT complex shows (A) Re-CsrA binding does not overlap with the Tir^32-80^ binding region and the C-terminus of CesT (residues I132-Y153) bind the same concave surface of CesT as Tir residues 65–75. Close up view of (B) the Re-CsrA and Tir^32-80^ binding region and (C) the second Tir binding region (residues 65–75) closely mimicked by CesT residues 132–153. Re-CsrA and CesT are coloured orange and green, respectively, with the C-terminus of CesT coloured cyan (residues 132–154) and red from the second CesT molecule in the dimer. Tir^32-80^ is coloured pink and CesT^138^ is coloured by secondary structural elements β-strands (purple), α-helices (blue), and loops (grey). The RMSD of the CesT alignment is 1.3 Å over 123 Cα atoms.

CesT and CesT^138^ form a dimer in solution (S2 Fig) consistent with previous reports [30], and is a property conserved among T3SS class I chaperones. Although only one molecule of CesT^138^ was present in the crystallographic asymmetric unit, the dimer interface is clearly present along the principle 2-fold axis of symmetry (S3A Fig). Recently, the structure of CesT in complex with CsrA was reported [28]. CesT in the CsrA-CesT complex also adopts the same dimer orientation as observed in the Tir^32-80^-CesT^138^ complex, providing further evidence that the domain swapped dimer of the previous unladen EHEC CesT structure is likely a crystallographic artifact (S3 Fig). Furthermore, structural alignment of Tir^32-80^-CesT^138^ to the CsrA-CesT complex reveals significantly different binding modes for Tir and CsrA to CesT (Fig 3A; a monomer of CesT is shown for simplicity). CsrA binds CesT predominantly through residue contacts along CesT α3 and α4 (red), in which the latter comes from the second molecule of the CesT dimer (Fig 3A). In contrast, Tir^32-80^ binds the cleft formed between CesT α1 and β1 (Fig 3A). CsrA doesn’t directly occlude binding of Tir residues 35–53 to CesT, but residues K26 and R31 come within very close proximity, 2.9 Å and 2.2 Å, from Tir residues G45 and S46, respectively (Fig 3B). The C-terminus of CesT (cyan) from the CsrA-CesT complex, which is absent from CesT^138^, self-associates by binding along the concave surface of CesT (Fig 3A and 3C). Furthermore, the C-terminus of CesT also forms α4 (red) that interacts with CsrA, locking the C-terminal CesT peptide (residues I32 to Y153) in place (Fig 3A and 3C). Interestingly, the CesT C-terminus occupies the same binding surface as Tir residues 65–75 (pink, Fig 3C), despite significantly different sequences. These findings likely explain why none of the Tir peptide-CesT complexes crystallized, as CesT residues I32 to Y153 would compete for the same binding grove as Tir residues 65–75, and thus required the truncation of the CesT C-terminus (CesT^138^). Furthermore, this explains why only CesT^138^ could co-purify with Tir^32-73^, as the self-associated C-terminus of CesT likely out-competes the smaller Tir peptide for binding along the same concave surface. Taken together, the structural data from the Tir^32-80^-CesT^138^ and CsrA-CesT complexes suggest that (*i*) CesT exists as an unswapped dimer, (*ii*) the Tir binding region of CesT exhibits significant plasticity that could accommodate the binding of multiple effectors with varying sequences, and (*iii*) the C-terminal extension of CesT is required for CsrA interaction that in turn could also prevent CesT from binding Tir.

### Identification of a second CesT-binding region at the carboxy-terminus of Tir

**Fig 4:**
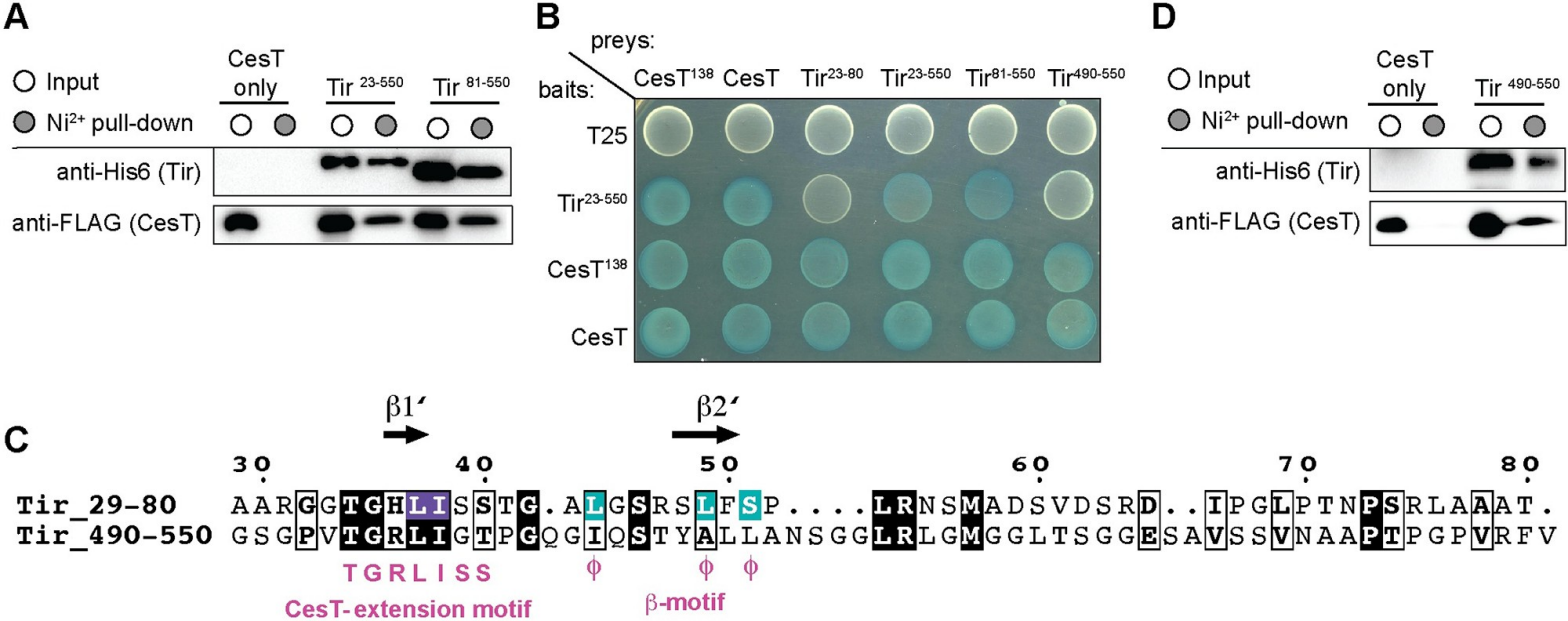
Identification of a second CesT-binding region in the carboxy-terminus of Tir. (A) His_6_-Tir^23-550^ and His_6_-Tir^81-550^, which lacks the CesT-binding region, were co-expressed with CesT-FLAG and the soluble cell lysates (input, white circle) and Ni^2+^ pull-down (elution, grey circle) fractions were analyzed by SDS-PAGE and immunoblotting for the ability of the Tir constructs to co-purify CesT-FLAG. (B) BTH101 reporter cells producing the indicated Tir and CesT constructs fused to the T18 or T25 domain fragments of the *Bordetella* adenylate cyclase were spotted on LB-agar supplemented with IPTG and X-gal. A positive protein interaction between the two fusion proteins is visualized by blue colony growth. (C) Tir-based sequence alignment to identify the second CesT-binding region at the carboxy-terminus of Tir. Residues highlighted in purple and cyan correlate to those in Fig 2D. Sequence alignment figures were generated using ESPript 3.0 [67]. (D) The His_6_-Tir^490-550^ peptide was co-expressed with CesT-FLAG and soluble cell lysate (input, white circle) and Ni^2+^ pull-down (elution, grey circle) fractions were analyzed by SDS-PAGE and immunoblotting for the ability of the isolated C-terminal Tir peptide to co-purify CesT-FLAG.

Tir^32-80^ binds the same hydrophobic surface in each monomer of the CesT^138^ dimer, producing a Tir^32-80^:CesT^138^ stoichiometry of 2:2 in solution, that was validated by gel filtration chromatography (S2 Fig). This crystal packing orientation was observed for the chaperone-effector fragment complexes of SycH-YscM2 [34], SycH-YopH [35], and ShcA-HopA1 [33]. However, gel filtration chromatography of the Tir^23-550^-CesT complex suggests that only a monomer of Tir^23-550^ binds a dimer of CesT (S2 Fig), consistent with a 1:2 Tir:CesT stoichiometry reported recently [36]. Since one molecule of Tir binds a dimer of CesT, but our crystallographic data suggest that two Tir^32-80^ fragments can bind a dimer of CesT, these data could be reconciled if full-length Tir contained a second uncharacterized CesT-binding region. Co-expression pull-down assays support this hypothesis as Tir^81-550^, which lacks the N-terminal CesT-binding region, retained the ability to pull-down CesT (Fig 4A). This was consistent with previous data showing that CesT can interact with N-terminal truncations of Tir in EHEC [37]. Furthermore, bacterial adenylate cyclase two hybrid (BACTH) assays showed that fusion of T18 to Tir^23-80^, Tir^23-550^, and Tir^81-550^ all had a positive interaction with CesT and CesT^138^ fused to T25 (blue colonies), but not to T25 alone (white colonies) (Fig 4B). We also observed CesT-CesT and Tir-Tir interactions in these assays consistent with previous reports [38, 39]. To identify the second CesT-binding region of Tir we used Tir residues 29–80 to conduct sequence alignments on a sliding window of ~50–80 amino acids. The carboxy-terminus of Tir (residues 490–550) had 20% sequence identity to Tir 29–80 and contained a TGRLIGT sequence similar to the sequence that forms β1’ in the N-terminal region of Tir (Fig 4C). T18 fused to Tir^490-550^ showed a strong interaction with CesT fused to T25 by BACTH assays (Fig 4B), and was able to pull-down CesT in co-expression pull-down experiments (Fig 4D), confirming this site as a second CesT-binding region. Taken together these data suggest that Tir is unique among *E*. *coli* effectors in that it contains a second carboxy-terminal CesT-binding region that is sufficient for interaction with CesT. Furthermore, both of the Tir CesT-binding regions have a conserved sequence motif distinct from the known chaperone binding β-motif.

### The C-terminal CesT-binding region of Tir is required for efficient Tir translocation and pedestal formation

**Fig 5:**
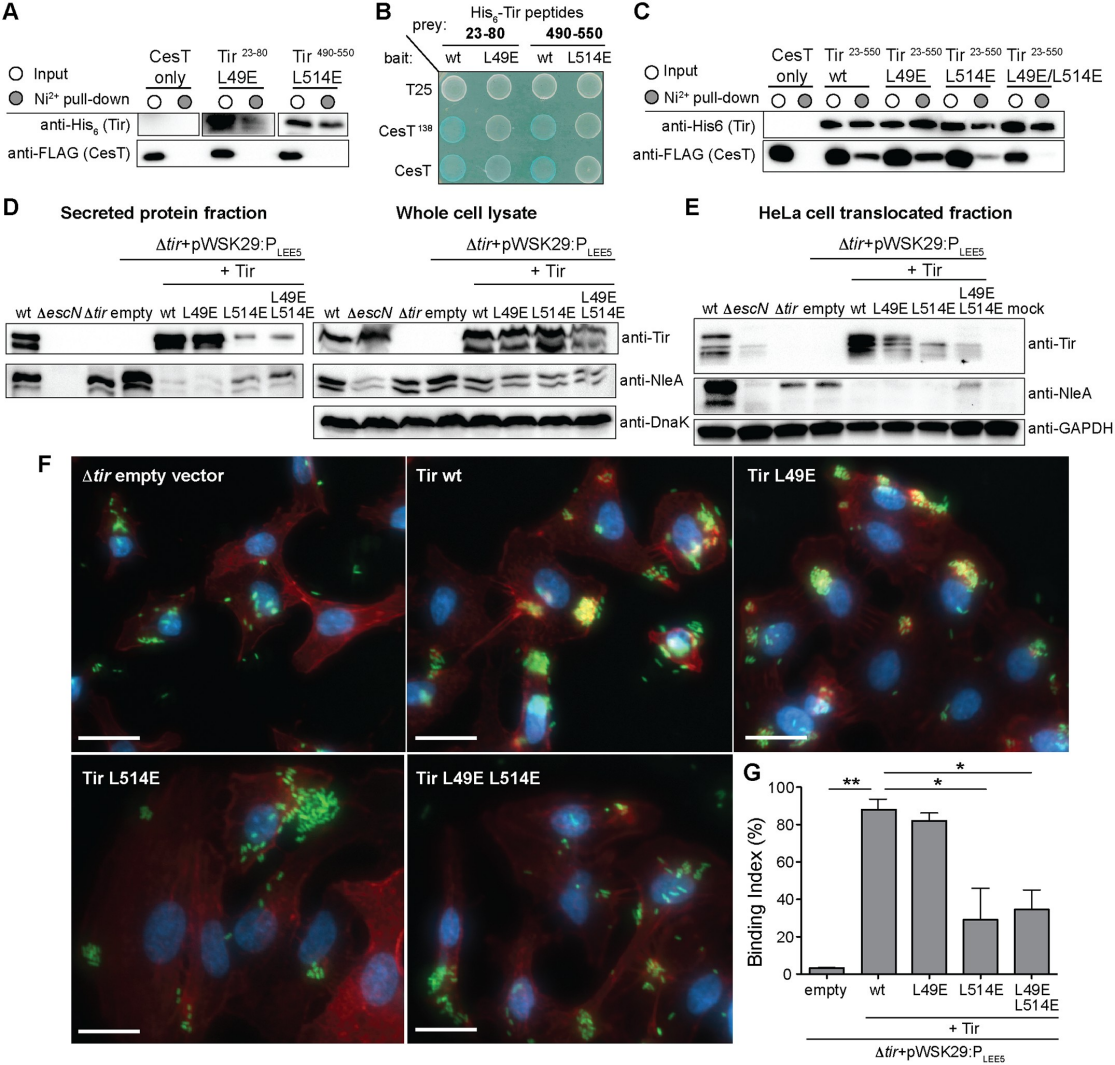
Disruption of the C-terminal CesT-binding region impairs Tir secretion, host translocation, and pedestal formation. (A) The His_6_-Tir^23-80^ L49E and His_6_-Tir^490-550^ L514E peptides were co-expressed with CesT-FLAG and the soluble cell lysates (input, white circle) and Ni^2+^ pull-down (elution, grey circle) fractions were analyzed by SDS-PAGE and immunoblotting for the ability of the Tir peptide β-motif variants to co-purify CesT-FLAG. (B) BTH101 reporter cells producing the indicated Tir and CesT constructs fused to the T18 or T25 domain fragments of the *Bordetella* adenylate cyclase were spotted on LB-agar plates supplemented with IPTG and X-gal. A positive protein interaction between the two fusion proteins is visualized by blue colony growth. (C) The His_6_-Tir^23-550^ L49E, His_6_-Tir^23-550^ L514E, and His_6_-Tir^23-550^ L49E L514E variants were co-expressed with CesT-FLAG and the soluble cell lysates (input, white circle) and Ni^2+^ pull-down (elution, grey circle) fractions were analyzed by SDS-PAGE and immunoblotting for the ability of the Tir β-motif variants to co-purify CesT-FLAG. EPEC strains (D) grown in T3SS inducing conditions and (E) used to infect HeLa cells were analyzed by immunoblotting of secreted and whole cell lysate, and translocated fractions, respectively. The Δ*tir* mutant was *trans*-complemented with pWSK29-P_LEE5_ and expressing Tir, Tir L49E, Tir L514E, and Tir L49E L514E. DnaK and GAPDH were used as loading controls. (F) Immunofluorescence microscopy of EPEC infected HeLa cells stained with phalloidin to detect F-actin (red), DAPI to detect nuclei (blue), and bacteria containing a GFP-plasmid (green). The Δ*tir* mutant strain complemented with pWSK29-P_LEE5_ and expressing Tir, Tir L49E, Tir L514E, and Tir L49E L514E were analyzed. Representative images of infected HeLa cells with each strain are shown with quantitation in (F). White scale bars represent 20 μm and error bars represent standard deviation of the mean. Statistical significance was calculated using a one-way ANOVA with Tukey’s multiple comparison test. **P = 0.0011, *P ≤ 0.05.

Previous studies on the *Salmonella* effectors SipA and SptP showed that disruption or deletion of the β-motif prevented chaperone binding and subsequent effector secretion through the T3SS [31, 40]. To probe the function of the N- and C-terminal CesT-binding regions in Tir, we constructed leucine to glutamate mutants, L49E and L514E, within the N- and C-terminal β-motifs (cyan, Fig 4C). Since we showed that Tir lacking the N-terminal CesT-binding region (Tir^81-550^) could still interact with CesT, we first tested if β-motif variants of the individual N- and C-terminal Tir-peptide fragments retained CesT binding. Despite CesT being abundant in the soluble lysate, the Tir^23-80^ L49E and Tir^490-550^ L514E peptide variants were unable to pull-down CesT indicating that disruption of either β-motif prevented capture by CesT (Fig 5A). Consistent with this, T18 fusions of the Tir^23-80^ L49E and Tir^490-550^ L514E β-motif variants also showed little to no interaction in the presence of CesT and CesT^138^ fused with T25 in BACTH assays (Fig 5B, white colonies). However, the wild type T18 fusions of Tir^23-80^ and Tir^490-550^ constructs interacted with CesT and CesT^138^ fused to T25 (Fig 5B, blue colonies). As a control, we tested if the individual Tir^23-550^ L49E and Tir^23-550^ L514E variants were stable and could pull-down CesT, as both individual variants still have an intact CesT-binding region. Tir^23-550^ L49E and Tir^23-550^ L514E were both able to pull-down CesT suggesting that the mutations did not alter the global stability and structure of Tir, and that the L49E and L514E variants only locally disrupt CesT binding to Tir at the N- and C-terminal CesT-binding regions, respectively (Fig 5C). However, the Tir^23-550^ L514E variant showed lower levels of CesT pull-down compared to wild-type and L49E Tir^23-550^. We also tested the Tir^23-550^ L49E L514E double variant, as we predicted that disrupting both the N- and C-terminal CesT-binding regions would disrupt interaction with CesT. Indeed, the Tir^23-550^ L49E L514E double variant was significantly impaired for the ability to pull-down CesT (Fig 5C). As a functional readout, we tested whether the individual CesT-binding regions were required for Tir secretion by complementing a Δ*tir* mutant with *tir L49E*, *tir L514E*, or the *tir L49E L514E* double mutant. As a positive control, Tir secretion was restored to wild type levels in the Δ*tir* strain complemented with *tir* under its native LEE5 promoter (Fig 5D and S4A Fig). The Tir-L49E variant, which retained the functional C-terminal CesT-binding region, also restored Tir secretion to wild-type levels (Fig 5D and S4 Fig). However, the Tir-L514E variant that retains the N-terminal CesT-binding region, but disrupts the C-terminal CesT-binding region, drastically reduced Tir secretion to levels similar to the Tir-L49E L514E double variant (Fig 5D and S4 Fig). To test whether these same phenotypes were observed for the biologically relevant process of effector translocation, we analyzed infected HeLa cells for effector translocation using these same strains. Similar to the secretion assays, complementation of the Δ*tir* strain with *tir* or *tir L49E* restored Tir secretion and resulted in the host-cell modification of tyrosine phosphorylation as observed by the upper band in Tir immunoblots (Fig 5E). The Δ*tir* strain complemented with *tir L514E* or *tir L49E L514E* significantly reduced host-cell translocation (Fig 5E). Considering that host tyrosine phosphorylation of Tir is important for actin polymerization within the infected cell, we analyzed infected HeLa cells for pedestal formation. Consistent with our phenotypic data, the Δ*tir* strain complemented with empty plasmid was impaired for pedestal formation, whereas the Tir and Tir L49E-complemented strains formed thick actin pedestals underneath microcolonies of bacteria in >90% of all analyzed cells (Fig 5F and 5G). Bacterial cells expressing Tir L514E or Tir L49E L514E were significantly impaired for the formation of actin pedestals (Fig 5F and 5G), which is in agreement with the host translocation results. As a secondary proxy for effector secretion and translocation, we also probed the levels of NleA, as it is highly dependent on free CesT available in the cell. We observed reduced levels of endogenous NleA secretion and host translocation in the Δtir strain complemented with plasmids carrying the *tir* wt, *L49E*, and *L514E* variants (Fig 5D and 5E), likely due to the higher cellular levels of Tir from *trans-*complementation that would reduce the levels of free CesT. However, the *tir L49E L514E* double mutant displayed higher levels of NleA secretion and translocation (Fig 5D and 5E), correlating with reduced binding of the Tir L49E L514E mutant to CesT (Fig 5C). Together these data suggest that the C-terminal CesT-binding region of Tir is required for efficient Tir secretion, host translocation, and the formation of actin pedestals.

### Chromosomal *tir* mutants differentially affect Tir and NleA secretion dynamics

**Fig 6:**
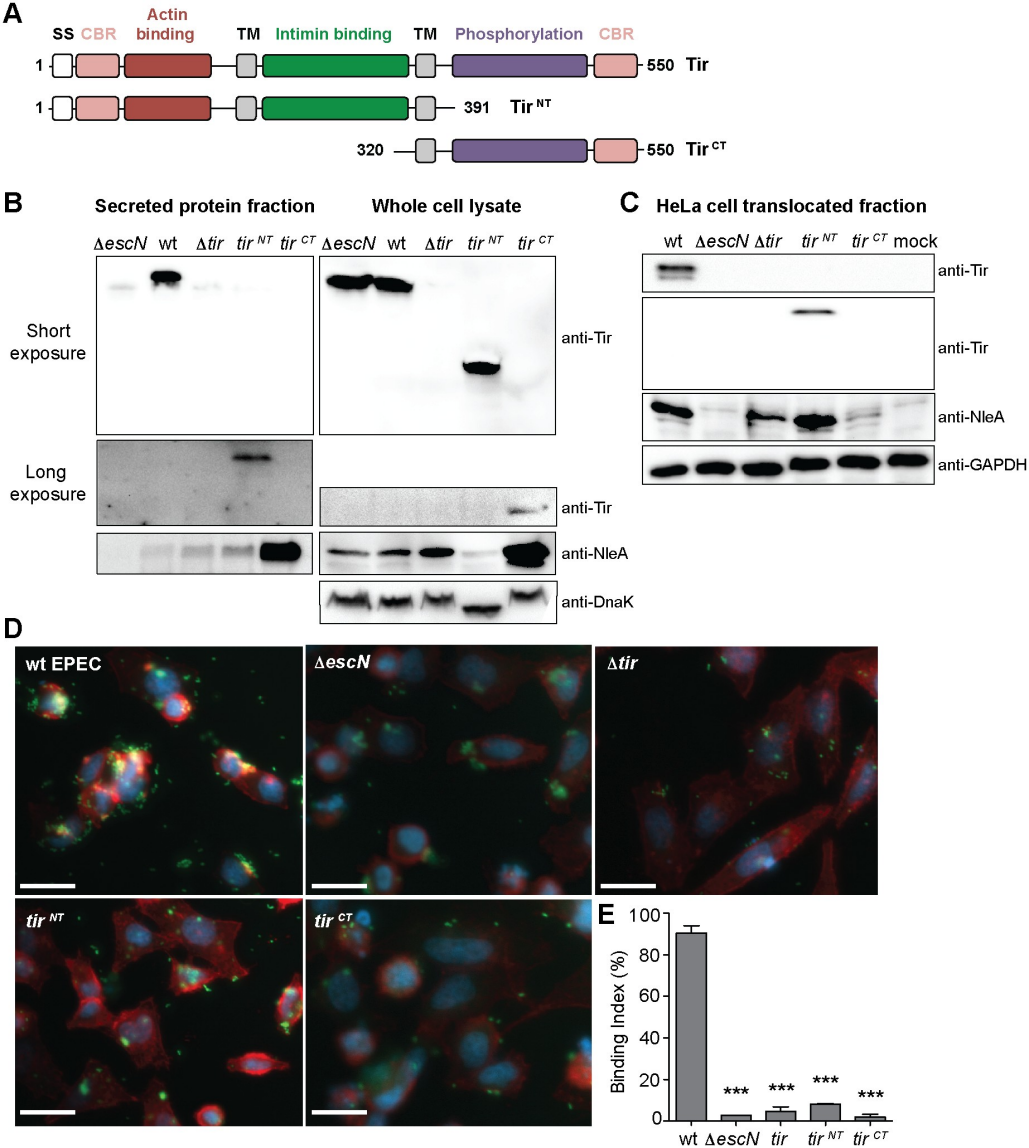
The C-terminal region of Tir is required for efficient secretion. (A) Schematic representation of Tir and the N- and C-terminal chromosomal truncation fragments. SS, secretion signal; CBR, CesT-binding region; TM, transmembrane domain. EPEC strains (B) grown in T3SS inducing conditions and (C) used to infect HeLa cells were analyzed by immunoblotting of secreted and whole cell lysate, and translocated fractions, respectively. DnaK and GAPDH were used as loading controls. (D) Immunofluorescence microscopy of EPEC infected HeLa cells stained with phalloidin to detect F-actin (red), DAPI to detect nuclei (blue), and bacteria containing a GFP-plasmid (green). Representative images of infected HeLa cells with each strain are shown with quantitation in (E). White scale bar represent 20 μm and error bars represent standard deviation of the mean. Statistical significance was calculated using a one-way ANOVA with Tukey’s multiple comparison test. ***P ≤ 0.0001.

Complementation studies showed that perturbation of CesT binding to the C-terminal region of Tir impaired Tir secretion and host translocation. Since the second CesT-binding region appears to be unique to Tir among all other CesT cargo, we tested the effect of N- and C-terminal domain truncations of Tir for secretion and translocation efficiency. Truncations of *tir* were constructed on the chromosome to produce Tir fragments encompassing residues 1–391 (*tir* ^*NT*^) and 320–550 (*tir* ^*CT*^) (Fig 6A). Tir ^NT^ contains the two elements predicted to be required for secretion, a T3SS signal sequence and a CesT-binding region. On the other hand Tir ^CT^ lacks a type III secretion signal sequence but contains the novel CesT-binding region in the C-terminal domain. Secretion assays conducted with the *tir* ^*NT*^ and *tir* ^*CT*^-expressing strains revealed that, although Tir ^NT^ was present at similar levels as full-length Tir in the bacterial cytosol, Tir ^NT^ was secreted at a very low level compared to full-length Tir (Fig 6B). This strain also displayed similar levels of NleA secretion but drastically reduced cytosolic levels of NleA compared to wild type (Fig 6B). These results are consistent with the Tir L514E variant that displayed impaired *in vitro* secretion (Fig 5D). The *tir* ^*CT*^ strain did not secrete Tir ^CT^ as expected, since it lacks a type 3 secretion signal, but it was present in the whole cell lysate albeit at a very low level compared to wild type Tir. Interestingly the *tir* ^CT^ strain displayed a marked increase in cytosolic and secreted NleA compared to wild type or *tir* ^*NT*^ strains (Fig 6B and S4B Fig). Next, we tested whether the *tir* mutant strains were functional for effector translocation into HeLa cells, and the formation of actin pedestals. Similar to the *in vitro* assays, the *tir* ^*NT*^ strain translocated lower levels of Tir ^NT^ compared to wild type EPEC, but had similar levels of NleA (Fig 6C). In contrast, the *tir* ^*CT*^ strain was unable to translocate Tir ^CT^ as expected, but now had low levels of NleA secretion similar to the Δ*tir* strain (Fig 6C). Other than wild type, none of the strains tested were able to form highly polymerized actin pedestals (Fig 6D and 6E). Taken together, these data indicate that the Tir C-terminal domain is important for Tir secretion and translocation into host cells, and that cellular levels of Tir in the bacterial cytosol affect the production and secretion of NleA.

### Identification of a CesT-extension motif that is required for high levels of effector translocation

**Fig 7:**
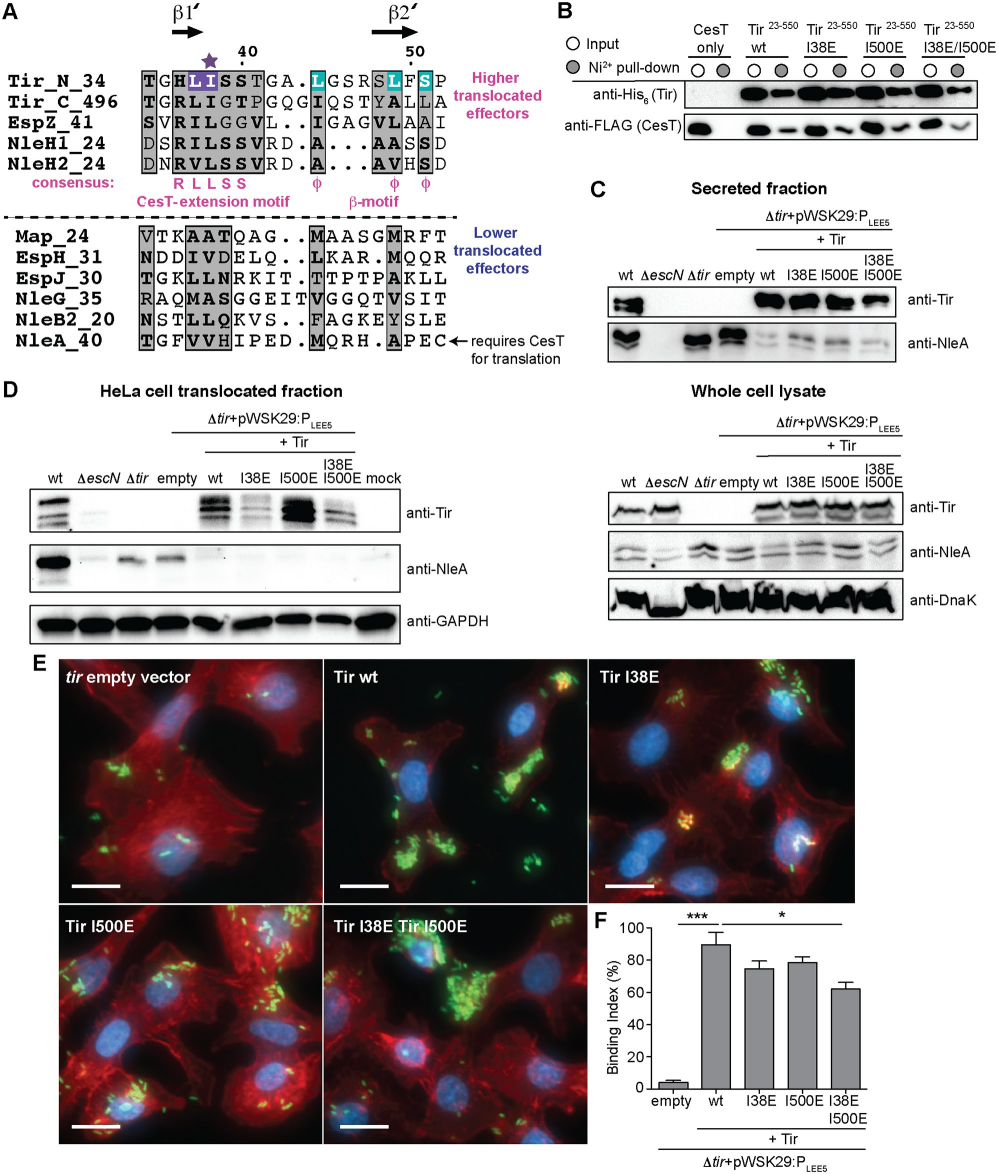
Disruption of the Tir CesT-extension motifs reduces host translocation and pedestal formation. (A) Sequence alignment of Tir with other T3SS effectors identifies the CesT-extension motif present within the CesT-binding regions of Tir and that of the highly translocated effectors NleH1, NleH2, and EspZ. Sequence alignment figures were generated using ESPript 3.0 [67], Φ represents the three hydrophobic residues found in the conserved β-motif, the purple star represents the residues mutated to glutamate, and residues highlighted in purple and cyan correlate to those in the Tir^32-80^ structure (Fig 2D). (B) The His_6_-Tir^23-550^ I38E, His_6_-Tir^23-550^ I500E, and His_6_-Tir^23-550^ I38E I500E variants were co-expressed with CesT-FLAG and the soluble cell lysates (input, white circle) and Ni^2+^ pull-down (elution, grey circle) fractions were analyzed by SDS-PAGE and immunoblotting for the ability of the Tir CesT-extension motif variants to co-purify CesT-FLAG. EPEC strains (C) grown in T3SS inducing conditions and (D) used to infect HeLa cells were analyzed by immunoblotting of secreted and whole cell lysate, and translocated fractions, respectively. The Δ*tir* mutant was *trans*-complemented with pWSK29-P_LEE5_ and expressing Tir, Tir I38E, Tir I500E, and Tir I38E I500E. DnaK and GAPDH were used as loading controls. (E) Immunofluorescence microscopy of EPEC infected HeLa cells stained with phalloidin to detect F-actin (red), DAPI to detect nuclei (blue), and bacteria containing a GFP-plasmid (green). The Δ*tir* mutant strain complemented with pWSK29-P_LEE5_ and expressing Tir, Tir I38E, Tir I500E, and Tir I38E I500E were analyzed. Representative images of infected HeLa cells with each strain are shown with quantitation in (F). White scale bar represents 20 μm and error bars represent standard deviation of the mean. Statistical significance was calculated using a one-way ANOVA with Tukey’s multiple comparison test. ***P ≤ 0.0001, *P ≤ 0.05.

Since the TGRLISS sequence in Tir was highly conserved between the N- and C-terminal CesT-binding regions (Fig 4C), we conducted a sequence search of this motif in other CesT cargo. We found a similar sequence, which we termed the CesT-extension motif, at the N-terminus of NleH1, NleH2, and EspZ (Fig 7A). The CesT-extension motif was much less conserved or not apparent in other CesT-dependent effectors (Fig 7A), suggesting that only a subset of CesT cargo contain this motif. We constructed disruptive glutamate variants in either the isoleucine or leucine residue present in the CesT-extension motif (residues starred purple in Fig 7A). This conserved isoleucine or leucine residue was chosen because, based on our structure, I38 in Tir^32-80^ extends into the hydrophobic CesT-binding pocket (purple, Fig 2D) and mutation to glutamate would disrupt the formation of β1’. Tir was tested first, showing that the individual Tir^23-550^ I38E and Tir^23-550^ I500E variants were stable and could pull-down CesT similarly to wild type Tir (Fig 7B). The Tir^23-550^ I38E I500E double variant was also able to pull-down CesT, albeit at slightly lower levels, suggesting that the CesT-extension motif is not obligatory for CesT binding (Fig 7B). To determine if the individual CesT-extension motifs were required for Tir secretion, the Δ*tir* strain was complemented with either *tir I38E*, *tir I500E*, or *tir I38E I500E* and Tir secretion was tested in secretion assays. Tir secretion was restored to wild type levels in the Δ*tir* strain complemented with *tir* under its native promoter and with the Tir-I38E and Tir-I500E single substitution variants (Fig 7C and S4C Fig). However, the Tir I38E I500E double variant had a slight reduction in Tir secretion compared to the wild type-complemented strain in these secretion assays (Fig 7C and S4C Fig). This is consistent with the reduced levels of CesT observed in the pull-down assays using the Tir I38E I500E double variant, but does not excluded the possibility that other factors contribute to the reduce secretion of the Tir I38E I500E variant. To further our *in vitro* secretion studies, we also analyzed infected HeLa cells for effector translocation and actin polymerization with the same strains. Complementation of the Δ*tir* strain with *tir*, *tir I38E*, and *tir I500E*, restored Tir translocation and tyrosine phosphorylation (Fig 7D), albeit Tir-I38E had slightly lower Tir levels. The Δ*tir* strain complemented with *tir I38E I500E* had the lowest levels of translocation and host modification (Fig 7D). Consistent with these results, we observed a significant reduction in actin pedestal formation in the strain expressing the Tir I38E I500E double variant (Fig 7E and 7F). We also probed the levels of NleA and similar to the Tir β-motif variants, we observed reduced levels of NleA secretion and translocation in the *tir* complemented strains (Fig 7C and 7D). However, the *tir* I38*E I500E* double mutant did not show higher levels of NleA secretion and translocation like the *tir* L49*E L514E* strain (Fig 7C and 7D), correlating with the pull-down data showing the Tir I38E I500E mutant retains CesT interaction (Fig 7B).

**Fig 8:**
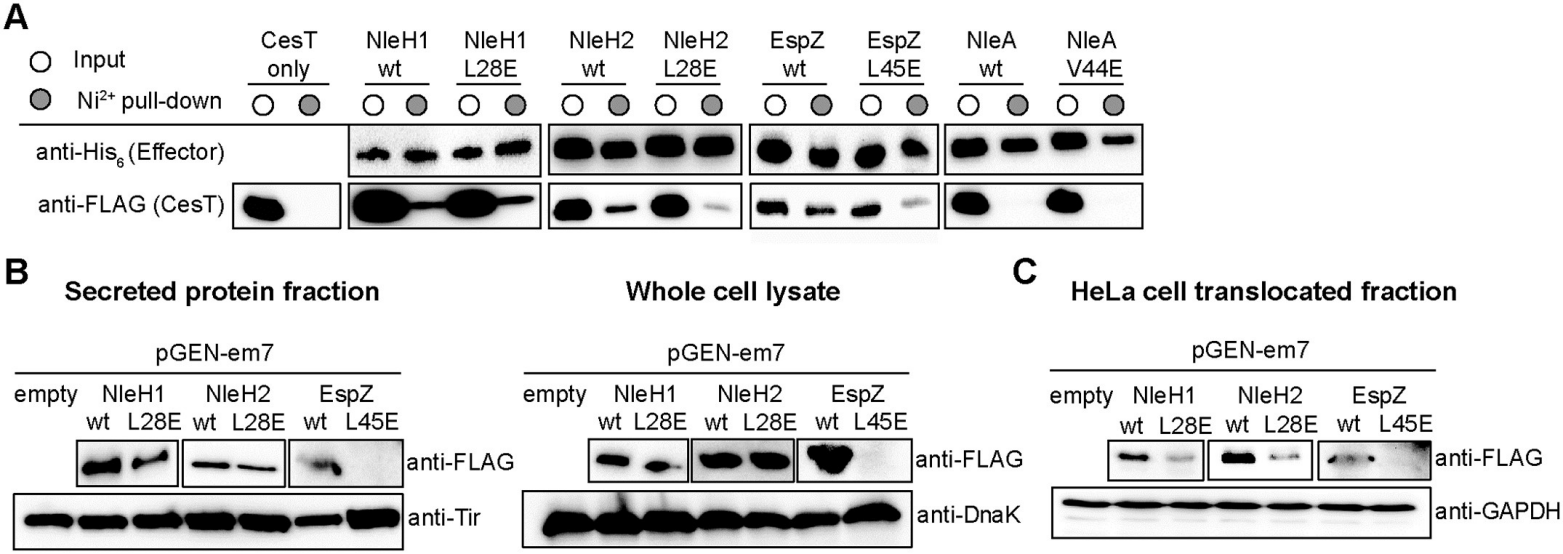
Disruption of the CesT-extension motif reduces host translocation of other EPEC effectors. (A) His_6_-tagged NleH1, NleH2, EspZ, and NleA and their respective CesT-extension motif variants were co-expressed with CesT-FLAG and the soluble cell lysates (input, white circle) and Ni^2+^ pull-down (elution, grey circle) fractions were analyzed by SDS-PAGE and immunoblotting for the ability of the effectors to co-purify CesT-FLAG. EPEC carrying the pGEN-em7 plasmid with various FLAG-tagged effectors and their respective CesT-extension motif variants (B) grown in T3SS inducing conditions and (C) used to infect HeLa cells were analyzed by immunoblotting of secreted and whole cell lysate, and translocated fractions, respectively. Tir, DnaK, and GAPDH were used as loading controls.

To expand these observations with other effectors, we made the equivalent glutamate substitutions in NleH1 (L28E), NleH2 (L28E), and EspZ (L45E). We also included NleA (V44E) in this analysis because it is a highly translocated effector but contains a very divergent sequence to the CesT-extension motif (Fig 7A). We tested if His_6_-tagged effectors and their putative CesT-extension motif variants were stable and could pull-down CesT. NleH1, NleH2, EspZ, and their respective glutamate variants were all able to pull-down CesT, however reduced CesT pull-down was observed for NleH2 L28E and EspZ L45E (Fig 8A). Interestingly, we observed little to no CesT in the NleA pull-downs, suggesting that CesT may not act as a chaperone for NleA but is only required to antagonize CsrA repression of the *nleA* 5’-UTR [27]. To determine if the CesT-extension motif affects secretion of these effectors (excluding NleA), secretion assays were conducted using EPEC carrying a plasmid expressing FLAG-tagged versions of NleH1, NleH2, EspZ, and their glutamate variants (Fig 8B). NleH1 L28E had a slight reduction in secretion and NleH2 L28E had little to no reduction in secretion compared to wild type. EspZ L45E was not detected in either the supernatant or whole cell lysate suggesting the mutation affected overall effector stability, possibly due to reduced CesT binding in the cytosol, which would be consistent with the pull-down data. Finally, we tested if the glutamate mutations in the CesT-extension motif of each effector affected translocation into infected HeLa cells. Under infection conditions, there was reduced translocation of NleH1 L28E, NleH2 L28E, and EspZ L45E compared to the wild type effectors (Fig 8C). Together these data suggest that the presence of a CesT-extension motif, in addition to the canonical β-motif, contribute to cargo recognition by CesT in a subset of effectors.

## Discussion

Tir drives the committal step of intimate attachment between EPEC and the host cell through an extracellular interaction with intimin on the bacterial cell surface [41, 42]. Thus, despite at least 12 effectors having full or partial dependence on CesT for translocation into host cells, Tir is the first effector to be released [22, 23]. This ultimately leads to attaching and effacing lesions from Tir-dependent signaling cascades that cause actin polymerization at the site of attachment, followed by Tir-independent effects on the host cell resulting from the release of secondary effectors [43]. Despite almost two decades of work on Tir and CesT, the mechanism that discriminates Tir secretion over that of other effectors remains unclear. Early transcriptional activation of *tir* is one possible mechanism to ensure Tir is available first for secretion. However, this does not seem to be the driving mechanism because LEE5 (which contains *tir*, *cesT*, and *eae*) is activated concurrently with LEE2, LEE3, LEE4, and LEE7 approximately 70 minutes after exposure to T3SS inducing conditions [44]. Furthermore, Tir secretion occurs approximately 30 minutes after transcriptional activation [44], suggesting a post-translational mechanism might drive preferential Tir secretion, as a number of other LEE-encoded effectors would also be present in the cytosol that would require discrimination within the cell. The nature in which Tir interacts with CesT is a possible mechanism by which this discrimination occurs. In this work we identified a second CesT-binding region in the C-terminal domain of Tir, and identify a CesT-extension motif, distinct from the known chaperone-binding β-motif, that is present in the CesT-binding regions of Tir and other highly translocated effectors. Our data raise the possibility that the presence of these features contribute to cytosolic discrimination by CesT, however formal assessment of this hypothesis in the context of the bacterial cell remains to be tested.

CesT has been the focus of structural and functional studies since its initial discovery as a Tir-specific chaperone [18]. Following structural determination of EHEC CesT [30], the domain swapped dimer has been a topic of debate as to whether it represents a biologically relevant conformation [45]. Our structural data on Tir^32-80^-CesT^138^, and work from others on the CsrA-CesT complex [28], indicates that CesT adopts the same dimer conformation even though it binds different substrates in separate locations. These data, along with solution state structural data from NMR [45], provide evidence that the domain swapped EHEC CesT dimer is most likely an artifact of crystallization, possibly arising from plasticity in the effector-binding region. Interestingly, the structure of the SrcA chaperone from *Salmonella* also exhibited plasticity in the effector-binding region [15], which suggests this could be a conserved property among multi-cargo chaperones to accommodate binding of multiple effectors.

Previous studies predicted that Tir residues 39–83 contained a degenerate CesT-binding domain [21]. Our Tir-peptide pull-down and structural data show that the minimal CesT-binding region of Tir is residues 35–75. In addition to providing the first structural view of an effector bound to CesT, the structure of the Tir^32-80^-CesT^138^ complex was critical in identifying the second CesT-binding region within the carboxy-terminus of Tir (residues 490–550). This chaperone binding arrangement appears unique to Tir, as there is no evidence for a secondary CesT-binding region in other CesT effector cargo. For example, the first 101 residues of Map were sufficient to interact with CesT, whereas the C-terminal 103 residues showed no interaction [20]. Furthermore, the structural data lead to the identification of a putative CesT-extension motif present in the N-terminal region of NleH1, NleH2, EspZ, and in both the amino- and carboxy-terminal regions of Tir. This region was so named because, according to our structure, it appears to extend the β-sheet core of CesT in its cargo-laden state. Mutation of the CesT-extension motif showed that it was most important for efficient host translocation. A commonality among effectors containing the CesT-extension motif (Tir, NleH1, NleH2, and EspZ) is that they are among the most highly translocated effectors among CesT cargo [23]. The presence of this motif in a subset of effectors (and its duplication in the case of Tir) may help to understand how multi-cargo chaperones recognize and possibly discriminate between effectors in the cell.

Efficient Tir-CesT complex formation is likely driven by the presence of two CesT-binding regions in Tir, however additional factors likely contribute to the observed preference for initial Tir secretion [21–23]. One possible contributing factor could be posttranslational modification of the C-terminus of CesT, which contains a site for tyrosine phosphorylation (Y152/153) [25]. A recent study showed that EPEC expressing a CesT Y153F variant exhibited a global increase in effector secretion, whereas EPEC expressing CesT Y152F was attenuated for NleA translocation into HeLa cells [26]. The latter result could be explained by the recent structure of CsrA in complex with CesT, which shows that CesT Y152 forms critical hydrogen bonds along the CsrA binding interface [28]. Therefore, CesT Y152F likely has reduced binding for CsrA and in turn is unable to depress the *nleA* 5’-UTR. We also observed that NleA had little to no binding of CesT in our pull-down assays. This suggests that the requirement of CesT for NleA secretion dynamics may be indirect and relate more to CsrA antagonism of the *nleA* 5’-UTR. This would be consistent with other work showing that NleA is only partially dependent on CesT for translocation into host cells [35]. Alternatively, it is possible that NleA or CesT may need post-translational modifications to facilitate interaction, require a third unknown co-chaperone, or NleA follows a chaperone-independent secretion pathway recently reported for a subset of *Shigella* T3SS effectors [46].

**Fig 9:**
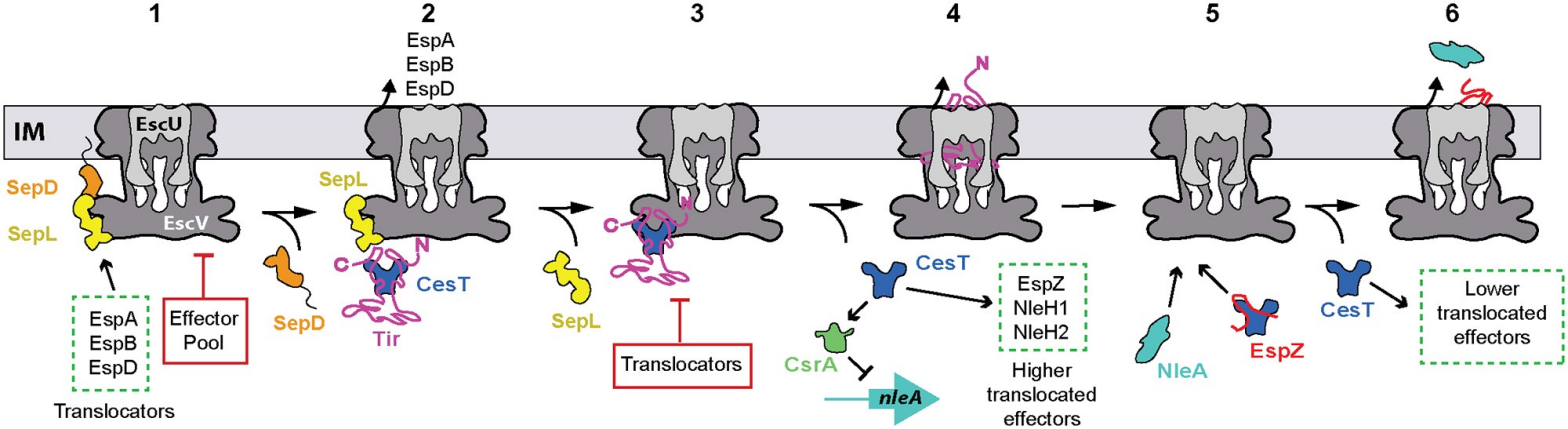
Working model for differential delivery of EPEC effectors. Stepwise progression of the affinity-switch model for T3SS substrate activity from translocators to late effectors. The ATPase-sorting platform complex, CesL, and EscP have not been included for simplicity. SepD, orange; SepL, yellow; EscU, light gray; EscV, dark grey; Tir, pink; CesT, blue; NleA, cyan; CsrA, green; EspZ red; IM, inner membrane. This figure is adapted from Portaliou et al. [36].

In addition to driving the formation of the Tir-CesT complex, the second CesT-binding region in Tir might stabilize a distinct conformation of the C-terminal domain of Tir that may be required for efficient targeting of the Tir-CesT complex to the T3SS sorting platform. Our data with the CesT-binding region mutants within the C-terminal domain of Tir (L514E variant), along with the chromosomal *tir* ^*NT*^ mutant (C-terminal domain truncation) support this possibility. For example, the Tir L514E and Tir ^NT^ constructs both contain a functional CesT-binding region and a type III secretion signal, but show significantly reduced secretion and translocation efficiency into HeLa cells. Furthermore, deletion of residues 519–524 in the C-terminal domain of EHEC Tir also showed significantly reduced Tir secretion [47]. Interestingly, EHEC Tir residues 519–524 align with EPEC Tir residues 511–516, which overlap with the predicted β-motif in the second CesT-binding region (ie. the Tir L514E mutant we tested). Recently, it was shown that an affinity switch controls substrate secretion hierarchy in the T3SS of EPEC. The SepL-SepD complex engages EscV (translocase) to ensure efficient targeting and secretion of the translocators, while simultaneously inhibiting effector targeting [36]. SepD release from the complex disrupts SepL-EscV crosstalk, leading to equivalent targeting of translocators and effectors for secretion. This is followed by the eventual release of SepL that results in inhibition of translocators and exclusive targeting of late effectors. This study also showed that the Tir-CesT complex had a two-fold increase in affinity for wild-type inner membrane vesicles that contain SepD, SepL, and EscV, over CesT alone [36]. This increased affinity for the Tir-CesT complex is probably due to a SepL-Tir interaction, which is supported by previous pull-down data in EHEC where the C-terminal 48 residues of SepL interact with Tir [37]. This particular Tir interaction site on SepL in EHEC also overlaps with one of the two EscV binding patches observed on EPEC SepL from peptide-binding array data [36]. Considering these studies with our findings, we provide an extended version of the affinity-switch model proposed by Portaliou et al. that includes differential secretion of late effectors (Fig 9). In this model, the SepD-SepL (and likely CesL) complex interacts with EscV and allows for strict translocator secretion (EspA, EspB, EspD) while simultaneously preventing effector secretion. It is also noteworthy that EscP binds SepL in a calcium-dependent manner and also contributes to the blocking of late effectors for secretion [48]. At this point it is plausible that CesT dimers are predominantly loaded with Tir in the cytosol since Tir contains two CesT-binding regions. After SepD dissociation from the EscV membrane complex, the Tir-CesT complex might compete with SepL for EscV binding. This competition could be mediated by the C-terminal domain of Tir leading to strict docking of the Tir-CesT complex to EscV over other effectors. Alternatively, the Tir-CesT complex may compete for EscP binding to SepL, leading to early docking of the complex within the sorting platform. Either possibility may explain preferential Tir release over other effectors, but it remains to be shown experimentally. SepL eventually dissociates from EscV, a process that may be directly influenced by Tir-SepL interactions, leading to the inhibition of translocator secretion and strict targeting of late effectors. Rapid release of Tir would result in the accumulation of free CesT in the cell, which in turn can antagonize CsrA repression of the *nleA* transcript through CesT-CsrA interactions, and increase binding of other highly translocated effectors such as EspZ, NleH1, and NleH2. Further depletion of the effector pool liberates more CesT, allowing for binding and secretion of lower translocated effectors.

## Material and methods

### Strains and cloning

Bacterial strains, plasmids, and primers used in this study are described in Tables A and B in SI Text. Phusion or Phire II polymerase (Thermo Fisher Scientific) were used for all PCR reactions, oligonucleotide primers were synthesized by Sigma, and site-directed mutants were constructed by using the Q5 site-directed mutagenesis kit (NEB) with the mutation encoded in the amplification primer. For protein expression, CesT and the C-terminal truncation construct encoding residues 2–138 (CesT^138^) were cloned into pET28a using the NheI/XhoI restriction sites. To allow for subsequent sub-cloning of CesT the internal NdeI site was removed by introducing a silent mutation in the coding region of His138 (CAT::CAC). CesT and CesT^138^ were then sub-cloned into the second multiple cloning site of pCOLADuet-1 using the NdeI/XhoI restriction sites. CesT-FLAG and CesT^138^-FLAG were also cloned into pCOLADuet-1 using the NdeI/XhoI site with the FLAG-tag encoded within the PCR amplification primer. Tir and EspZ; NleH1 and NleH2; and NleA constructs were cloned using the BamHI/HindIII, BamHI/SalI, and BamHI/NotI sites, respectively, into the first multiple cloning site of pCOLADuet-1 with CesT or CesT^138^ (and/or their FLAG-tag versions) in the second multiple cloning site for co-expression and pull-down experiments. For T3SS complementation assays, the LEE5 promoter encompassing nucleotides -7 to -323 from the translational start site of *tir* was cloned into pWSK29 using the XhoI/HindIII restriction sites. Subsequently, *tir* was cloned into the pWSK29-P*tir* plasmid using the HindIII/NotI restriction sites. For effector secretion studies, effectors carrying a C-terminal FLAG-tag encoded in the primer were cloned into pGEN-*luxCDABE* using the SnaBI/SacI sites. For BACTH experiments, CesT and Tir constructs were cloned into the pKNT25 and/or pUT18C plasmids using the XbaI/SacI sites. All plasmids were verified by sequencing.

### Antibodies and immunoblotting

Protein fractions were separated by SDS-PAGE and transferred to a polyvinylidene fluoride membrane, and blocked in Tris-buffered saline with 0.1% (w/v) Tween (TBST) containing 5% skim milk. Membranes were then probed using the following primary antibodies: mouse monoclonal anti-Tir (1:2000) for full-length and C-terminal fragments, rat polyclonal anti-Tir from *C*. *rodentium* (1:2000) [49] for N-terminal fragments, rat polyclonal anti-NleA from EHEC (1:2000) [50], mouse α-DnaK (Stressgen, 1:5000), mouse α-FLAG M2 (Sigma, 1:5000), mouse α-His6 (GE Healthcare, 1:3000), or goat α-GAPDH (R&D Systems Inc., 1:5000). The blots were then developed using the following secondary antibodies: goat anti-mouse (1:5000, Jackson), goat anti-rat (1:2000, EMD Millipore), or donkey α-goat (Santa Cruz Biotechnology, 1:5000) conjugated to horseradish peroxidase, and imaged using the Clarity Western ECL (BioRad) or SuperSignal West Femto Maximum Sensitivity (ThermoFisher) substrates and a ChemiDoc XRS+ (BioRad).

### Co-expression and pull-down experiments

*E*. *coli* BL21-CodonPlus (DE3) cells were transformed with the appropriate co-expression plasmid (pCOLADuet-1 containing N-terminal His6-tagged effector and C-terminal FLAG-tagged CesT), grown overnight in LB media with 50 μg/mL kanamycin at 37°C with shaking, sub-cultured 1:50 in 50 mL LB media with 50 μg/mL kanamycin to an OD_600_ of ~0.4–0.5, and moved to 30°C. When the cultures reached OD_600_ of ~0.6–0.7 protein expression was induced by the addition of isopropyl-D-1-thiogalactopyranoside (IPTG) to a final concentration of 0.5 mM. The cells were incubated for 3 h at 30°C, harvested by centrifugation at 5000 *g* for 10 min, and frozen on dry ice. Cell pellets were thawed and re-suspended in 2 mL of lysis buffer (50 mM Tris-HCl pH 7.5, 300 mM NaCl, 20 mM imidazole, 5% (v/v) glycerol, 2 mM 2-mercaptoethanol). Re-suspended cells were lysed by sonication and cell debris was removed by centrifugation at 16000 *g* for 30 min. The resulting supernatant was passed over a gravity column containing 0.2 mL Ni-nitrilotriacetic acid (NTA) agarose resin (Qiagen) that was pre-equilibrated with lysis buffer. Bound protein was washed with 100 column volumes of lysis buffer, and eluted with 5 column volumes of lysis buffer with 250 mM imidazole. Soluble lysate and elution fraction samples were mixed with equal parts of 2X SDS-PAGE loading dye and analyzed by SDS-PAGE and western blotting.

### Protein expression and purification

The following protocol was used to express and purify CesT, CesT^138^, and all the Tir-CesT complexes for crystallization. *E*. *coli* BL21-CodonPlus (DE3) cells were transformed with the appropriate plasmid, grown overnight in LB media with 50 μg/mL kanamycin at 37°C with shaking, sub-cultured 1:50 into 1 L LB media with 50 μg/mL kanamycin to an OD_600_ of ~0.4–0.5, and moved to 18°C. When the cultures reached OD_600_ of ~0.6–0.7 protein expression was induced by the addition of IPTG to a final concentration of 0.5 mM. The cells were incubated overnight at 18°C, harvested by centrifugation at 5000 *g* for 10 min, and frozen on dry ice. Cell pellets were thawed and re-suspended in 25 mL of lysis buffer (50 mM Tris-HCl pH 7.5, 300 mM NaCl, 10 mM imidazole, 5% (v/v) glycerol, 2 mM 2-mercaptoethanol, and one complete mini protease inhibitor cocktail tablet (Roche)). Re-suspended cells were lysed by sonication and cell debris was removed by centrifugation at 31000 *g* for 30 min. The resulting supernatant was passed over a gravity column containing 3 mL Ni-NTA agarose resin (Qiagen) that was pre-equilibrated with lysis buffer. Bound protein was washed with 10 column volumes of lysis buffer, 3 column volumes lysis buffer with 20 mM imidazole, and eluted with 5 column volumes of lysis buffer with 250 mM imidazole. The eluted fraction was concentrated using a 10 or 30 kDa cut-off Amicon ultrafiltration device (EMD Millipore) and further purified and buffer exchanged into 20 mM Tris-HCl pH 7.5 and 150 mM NaCl by size exclusion chromatography using a HiLoad 16/60 Superdex 200 prep-grade gel-filtration column (GE Healthcare). The purified constructs were >95% pure as judged by SDS-PAGE and stable for at least 1 week at 4°C.

### Crystallization, data collection, and structure determination

Purified His_6_-Tir^32-80^-CesT^138^ was concentrated to ~14 mg/mL and screened for crystallization conditions at 22°C using hanging-drop vapour diffusion in 24-well VDXm plates (Hampton Research) and the MCSG 1–4 sparse matrix suites (Anatrace). The best initial crystallization hits were obtained from MCSG-1 condition #17 and MCSG-3 condition #44. Optimized crystals were grown by mixing 2 μL of 14 mg/mL His6-Tir^32-80^-CesT^2-138^ with 1.5 μL of precipitant solution (0.1 M Tris-HCl pH 7.5, 0.2 M MgCl_2_, and 17% (w/v) PEG3350) equilibrated against 500 μL of 1.7 M MgSO_4_. The crystals took 1–3 weeks to reach maximum size and were frozen without cryoprotection in liquid nitrogen. Diffraction data were collected at a wavelength of 0.98 Å on beam line 08B1-1 at the Canadian Light Source (CLS) (Table 1). The data were indexed and integrated with iMosflm [51] and scaled using SCALA in the CCP4i suite [52]. The structure was determined by molecular replacement with Phenix Phaser [53] using EHEC CesT residues 38–131 (PDB ID: 1K3E) as a search model. The resulting electron density map enabled Phenix AutoBuild [54] to build ~70% of CesT^138^. The remaining CesT^138^ residues and the Tir^32-80^ fragment were built manually in Coot [55] and alternated with refinement using phenix.refine [56]. Translation/Libration/Screw (TLS) groups were used during refinement and determined automatically using the TLSMD web server [57, 58]. Structure figures were generated using PYMOL Molecular Graphics System (DeLano Scientific), and quantitative electrostatics were calculated using PDB2PQR [59, 60] and APBS [61].

### Analytical size exclusion chromatography

Elution fractions from various Tir-CesT co-expression pull-down experiments were concentrated to 100 μL, applied to a Superdex 200 10/300 GL column, and eluted using 20 mM Tris-HCl pH 7.5 and 150 mM NaCl. Protein standards used to calibrate the column were ferritin (440 kDa), conalbumin (75 kDa), ovalbumin (44 kDa), ribonuclease A (13.7 kDa), and aprotinin (6.5 kDa).

### BACTH assays

*E*. *coli* BTH101 cells were co-transformed with the various pKNT25 and pUT18C based plasmids and recovered on LB-Kan^50^-Amp^100^ agar plates at 37°C. Single colonies were grown overnight with shaking at 37°C in LB-Kan^50^-Amp^100^, and then 20 μL of each sample was spotted onto LB agar plates containing Kan^50^, Amp^100^, 0.5 mM IPTG, and 40 μg/mL 5-bromo-4-chloro-3-indolyl-β-D-galactopyranoside (X-gal). Plates were incubated for 24–48 h at 30°C for the development of blue colonies.

### Construction of chromosomal deletions

Primer pairs with 48 nucleotide homologous tails to *escN* or *tir* were used to amplify linear PCR products with pKD3 for generation of the *escN* or various *tir* mutants. In-frame marked mutants of EPEC replacing *escN* residues 9–446 or *tir* residues 50–319, 392–535, and 17–535 with chloramphenicol acetyltransferase (*cat*) were constructed using one-step λ-red inactivation with pKD46 and the transformed linear PCR products [62]. The *cat* cassette was then removed using plasmid pFLP2 and sucrose selection. All *tir* and *escN* deletions were verified by sequencing.

### Type III secretion assays

Secretion assays were performed similar to those described previously [63]. Standing overnight EPEC cultures grown in LB media (plus 100 μg/mL Amp as needed) at 37°C were sub-cultured 1:40 into 4 mL of pre-warmed Dulbecco’s modified eagle medium (DMEM) plus 2 mM ethylene glycol-bis(β-aminoethylether)-N,N,N',N'-tetraacetic acid (EGTA) in glass tubes. The cultures were incubated standing for 6 h at 37°C in a 5% CO_2_ incubator (OD_600_ of 0.7–0.9). The cultures were then harvested by centrifugation at 10000 *g* for 5 min, and the bacterial pellets were washed once in phosphate-buffered saline (PBS) and re-suspended in 1X SDS-PAGE loading dye (normalized by OD_600_ as necessary). The culture supernatant was passed through a low-protein binding 0.2 μm filter (Pall), and 1.35 mL aliquots were mixed with 150 μL of ice-cold 100% (w/v) trichloroacetic acid and incubated overnight at 4 °C. The solutions were centrifuged at 16000 *g* for 30 min, the supernatant was discarded, and the pellet was washed with 1 mL of ice-cold acetone. The washed pellets were centrifuged at 16000 *g* for 30 min, the pellet was air dried, and then re-suspended in 10 μL 1X SDS-PAGE loading buffer (or normalized by OD_600_ as necessary). Samples were then analyzed by SDS-PAGE using coomassie blue G250 stain or by western blotting.

### HeLa cell infections

HeLa cells (Coombes lab collection) were grown in DMEM + 10% fetal bovine serum at 37°C in a 5% CO_2_ incubator. Cells were routinely grown in 75 mm^2^ dishes (VWR) until confluent, and were then seeded at 2.2×10^6^ into 100 mm dishes (Corning) and incubated overnight. Prior to infection the HeLa cells in 100 mm dishes were washed with 10 ml of warm PBS. EPEC cultures were grown overnight standing at 37°C, harvested by centrifugation, and resuspended in DMEM. HeLa cells were then infected with EPEC at a multiplicity of infection of 50:1 for 3 h at 37°C in a 5% CO_2_ incubator. The cells were washed five times with cold PBS, harvested with a cell scraper, centrifuged at 1000 *g* for 5 min, and resuspended in 250 μL PBS + 0.5% (v/v) Triton X-100. Cells were then lysed for 30 min on ice with gentle rocking, centrifuged at 10000 *g* for 5 min, and the following supernatant was mixed with equal parts of 2X SDS-PAGE loading buffer for SDS-PAGE and western blot analysis.

### F-actin pedestal assays and immunofluorescence microscopy

HeLa cells maintained in DMEM + 10% fetal bovine serum were seeded at 1×10^5^ into 24 well tissue culture plates (VWR) containing 12 mm circle micro coverglass slips (VWR) and incubated overnight at 37°C in a 5% CO_2_ incubator. Prior to EPEC infection the glass slips were washed with 1 ml of warm PBS. EPEC cultures carrying a GFP expression plasmid for visualization (pFPV25.1 for the *tir* chromosomal domain mutants and pACYC-GFP for the *tir* complementation strains with various Tir point mutants) were grown standing overnight in LB media at 37°C, harvested by centrifugation, and resuspended in DMEM. HeLa cells were then infected with EPEC at a multiplicity of infection of 50:1 for 3 h at 37°C in a 5% CO_2_ incubator. Infected cells were washed with PBS (and after each subsequent step), fixed with 4% paraformaldehyde in PBS for 15 min, permeabilized with 0.3% (v/v) Triton X-100 in PBS for 5 min, and blocked with 5% (w/v) bovine serum albumin (BSA) in PBS for 30 min. F-actin was then stained using Alexa Fluor 568 Phalloidin (1:500, ThermoFisher) in PBS containing 1% (w/v) BSA for 60 min. Stained coverslips were washed in PBS and mounted on glass slides using ProLong Gold Antifade Mountant with 4’,6-Diamidino-2-phenylindole dihydrochloride (DAPI) for nuclear staining (Life Technologies) and allowed to sit overnight before sealing with nail polish. Microscopy was performed using a ZEISS Axio Imager 2 with 40X and 100X oil-immersion lenses with laser excitation. Images were captured using a Hamamatsu ORCA-R^2^ digital CCD camera and exported TIFF files were processed into their individual and composite color channels using ImageJ2 [64]. Quantification of pedestal formation (binding index) was conducted as described previously [65], where the percentage of infected HeLa cells that contained a microcolony of at least five GFP-positive bacteria associated with F-actin condensation were enumerated (co-localization, yellow).

## Supporting information

S1 Text Table A and B containing the strains, plasmids, and primers used in this study. (DOCX)

S1 Fig SDS-PAGE analysis of the Tir peptide-CesT complexes. SDS-PAGE analysis of His_6_-Tir peptides co-expressed and purified with (A) CesT and (B) CesT^138^. The resolved samples represent elution fractions from Ni-affinity pull-downs, with Tir residues labeled on top of the corresponding lanes.
(TIF)

S2 Fig Gel filtration chromatography of the purified Tir-CesT complexes. The Tir^23-550^-CesT (black) and Tir^81-550^-CesT (green) complexes elutes at ~180–200 kDa. The Tir^23-80^-CesT (pink), Tir^32-80^-CesT (red), Tir^35-77^-CesT (purple), and Tir^490-550^-CesT (orange) complexes elute at ~50 kDa. CesT (blue) and CesT^138^ (cyan) are shown for reference and elute as dimers at ~36 kDa. Arrows represent molecular weight standards that include ferritin, 440 kDa; conalbumin, 75 kDa; ovalbumin, 44 kDa, ribonuclease A, 13.7 kDa; and aprotinin, 6.5 kDa.
(TIF)

S3 Fig Structural comparison of Tir^32-80^-CesT^138^ and EHEC CesT dimers. Cartoon representation of the (A) Tir^32-80^-CesT^138^ dimer present along the crystallographic 2-fold axis of symmetry, and superposition with (B) EHEC CesT dimer, and (C) crystallographic symmetry mates of EHEC CesT whose domain swapped region superimposes with the Tir binding site. The bottom panel is rotated by 90° outwards. CesT^138^ is coloured purple (β-strands), blue (α-helices), and grey (loops); Tir^32-80^ is coloured pink; EHEC CesT is coloured green with the domain swapped region light green; and the EHEC CesT symmetry mates are coloured orange with the domain swapped region light green.
(TIF)

S4 Fig SDS-PAGE analysis of *in vitro* T3SS assays for the various Tir mutagenesis and domain mutants. EPEC strains grown in T3SS inducing conditions were analyzed for total secreted protein by SDS-PAGE for the (A) Tir β-motif variants, (B) Tir chromosomal truncation mutants, and (C) Tir CesT-extension motif variants. The gels were stained with coomassie brilliant blue G250.
(TIF)
