# Supplementary material for: Molecular basis for CesT recognition of type III secretion effectors in enteropathogenic Escherichia coli
Source: PLoS Pathog. 2018 Aug 17;14(8):e1007224. doi: 10.1371/journal.ppat.1007224 (PMC6114900; doi:10.1371/journal.ppat.1007224)
Supplement: S1 Text — (DOCX) [file ppat.1007224.s001.docx]

**Supporting Information**

**Molecular basis for CesT recognition of type III secretion effectors in enteropathogenic *Escherichia coli***

Dustin J. Little^1^ and Brian K. Coombes^1*^

^1^Department of Biochemistry & Biomedical Sciences, Michael G. DeGroote Institute for Infectious Disease Research, McMaster University, ON, Canada

*To whom correspondence should be addressed: Brian K. Coombes, Tel.: 905-525-9140 ext. 22454, E-mail: [coombes@mcmaster.ca](mailto:coombes@mcmaster.ca)

**Table A.** **Strains and plasmids used in this study.**

| Strain, plasmid, or primer | Description or characteristics | Source or reference |
| --- | --- | --- |
| Strains EPEC E2348/69 | *Enteropathogenic E. coli* isolate O127:H6 str. E2348/69 (Str^r^) | Brett Finlay [67] |
| EPEC Δ*cesT* | EPEC E2348/69 Δ*cesT*, T3SS deficient strain | Brett Finlay [18] |
| EPEC Δ*sepL* | EPEC E2348/69 Δ*sepL*, T3SS deficient strain | Brett Finlay [62] |
| EPEC Δ*escN* | EPEC E2348/69 Δ*escN*, T3SS deficient strain | This study |
| EPEC Δ*tir* | EPEC E2348/69 Δ*tir,* in-frame deletion of residues 17-535 | This study |
| EPEC Δ*tir-CT* | EPEC E2348/69 Δ*tir-CT*, in-frame deletion of residues 50-319 that produces a C-terminal fragment of Tir containing residues 320-550 | This study |
| EPEC Δ*tir-NT* | EPEC E2348/69 Δ*tir-NT*, in-frame deletion of residues 392-535 that produces an N-terminal fragment of Tir containing residues 1-391 | This study |
| TOP10 | *E. coli* cloning strain: F^-^ *mcr*A Δ(*mrr-hsd*RMS-*mcr*BC) φ80*lac*ZΔM15 Δ*lac*X74 *rec*A1 *ara*D139 Δ(*ara-leu*) 7697 *gal*U *gal*K *rps*L (Str^r^) *end*A1 *nup*G λ- | Invitrogen |
| BL21 CodonPlus (DE3) | *E. coli* expression strain: F^-^ *omp*T *hsd*S(r_B_^-^ m_B_^-^) *dcm*^+^ Tet^r^ *gal* λ(DE3) *end*A [*arg*U *pro*L Cam^r^] | Stratagene |
| BTH101 | *E. coli* BACTH strain: F-, cya-99 , araD139, galE15, galK16, rpsL1 (Str^r^), hsdR2, mcrA1, mcrB1 | Euromedix |
| Plasmids  pET28a | Expression vector | Novagen |
| pCOLADuet-1 | Co-expression vector | Novagen |
| pACYC184 | Low copy-number cloning vector | NEB |
| pWSK29 | Low copy-number cloning vector | [68] |
| pUT18C | BACTH plasmid, N-terminal T18 chimera | Euromedix |
| pKNT25 | BACTH plasmid, C-terminal T25 chimera | Euromedix |
| pET28-CesT | CesT expression plasmid, N-terminal His-6 tag | This study |
| pET28-CesT^*^ | CesT expression plasmid, contains silent mutation to remove internal NdeI site, N-terminal His-6 tag | This study |
| pET28-CesT^138^ | CesT 2-138 expression plasmid, N-terminal His-6 tag | This study |
| pCOLADuet-Tir^23-550^-CesT | Co-expression plasmid for N-terminal His-6 tagged Tir 23-550 and CesT | This study |
| pCOLADuet-Tir^81-550^-CesT | Co-expression plasmid for N-terminal His-6 tagged Tir 81-550 and CesT | This study |
| pCOLADuet-Tir^23-80^-CesT | Co-expression plasmid for N-terminal His-6 tagged Tir 23-80 and CesT | This study |
| pCOLADuet-Tir^23-80^-CesT^138^ | Co-expression plasmid for N-terminal His-6 tagged Tir 23-80 and CesT 2-138 | This study |
| pCOLADuet-Tir^32-80^-CesT | Co-expression plasmid for N-terminal His-6 tagged Tir 32-80 and CesT | This study |
| pCOLADuet-Tir^32-80^-CesT^138^ | Co-expression plasmid for N-terminal His-6 tagged Tir 32-80 and CesT 2-138 | This study |
| pCOLADuet- Tir^35-77^-CesT | Co-expression plasmid for N-terminal His-6 tagged Tir 35-77 and CesT | This study |
| pCOLADuet- Tir^35-77^-CesT^138^ | Co-expression plasmid for N-terminal His-6 tagged Tir 35-77 and CesT 2-138 | This study |
| pCOLADuet-Tir^32-73^- CesT | Co-expression plasmid for N-terminal His-6 tagged Tir 32-73 and CesT | This study |
| pCOLADuet-Tir^32-73^-CesT^138^ | Co-expression plasmid for N-terminal His-6 tagged Tir 32-73 and CesT 2-138 | This study |
| pCOLADuet-Tir^37-73^- CesT | Co-expression plasmid for N-terminal His-6 tagged Tir 37-73 and CesT | This study |
| pCOLADuet-Tir^37-73^-CesT^138^ | Co-expression plasmid for N-terminal His-6 tagged Tir 37-73 and CesT 2-138 | This study |
| pCOLADuet-Tir^490-550^-CesT | Co-expression plasmid for N-terminal His-6 tagged Tir 490-550 and CesT | This study |
| pCOLADuet-MCS2-CesT_FLAG | Co-expression plasmid for expressing only CesT with a C-terminal FLAG tag | This study |
| pCOLADuet-MCS2-CesT^138^_FLAG | Co-expression plasmid for expressing only CesT 2-138 with a C-terminal FLAG tag | This study |
| pCOLADuet-Tir^23-550^-CesT_FLAG | Co-expression plasmid for N-terminal His-6 tagged Tir 23-550 and CesT with a C-terminal FLAG tag | This study |
| pCOLADuet-Tir^23-550 L49E^-CesT_FLAG | Co-expression plasmid for N-terminal His-6 tagged Tir 23-550 L49E and CesT with a C-terminal FLAG tag | This study |
| pCOLADuet-Tir^23-550 L514E^-CesT_FLAG | Co-expression plasmid for N-terminal His-6 tagged Tir 23-550 L514E and CesT with a C-terminal FLAG tag | This study |
| pCOLADuet-Tir^23-550 L49E L514E^-CesT_FLAG | Co-expression plasmid for N-terminal His-6 tagged Tir 23-550 L49E L514E and CesT with a C-terminal FLAG tag | This study |
| pCOLADuet-Tir^23-550 I38E^-CesT_FLAG | Co-expression plasmid for N-terminal His-6 tagged Tir 23-550 I38E and CesT with a C-terminal FLAG tag | This study |
| pCOLADuet-Tir^23-550 I500E^-CesT_FLAG | Co-expression plasmid for N-terminal His-6 tagged Tir 23-550 I500E and CesT with a C-terminal FLAG tag | This study |
| pCOLADuet-Tir^23-550 I38E I500E^-CesT_FLAG | Co-expression plasmid for N-terminal His-6 tagged Tir 23-550 I38E I500E and CesT with a C-terminal FLAG tag | This study |
| pCOLADuet-Tir^81-550^-CesT_FLAG | Co-expression plasmid for N-terminal His-6 tagged Tir 81-550 and CesT with a C-terminal FLAG tag | This study |
| pCOLADuet-Tir^23-80^-CesT_FLAG | Co-expression plasmid for N-terminal His-6 tagged Tir 23-80 and CesT with a C-terminal FLAG tag | This study |
| pCOLADuet-Tir^23-80^-CesT^138^_FLAG | Co-expression plasmid for N-terminal His-6 tagged Tir 23-80 and CesT 2-138 with a C-terminal FLAG tag | This study |
| pCOLADuet-Tir^23-80_L49E^-CesT_FLAG | Co-expression plasmid for N-terminal His-6 tagged Tir 23-80 L49E and CesT with a C-terminal FLAG tag | This study |
| pCOLADuet-Tir^32-80^-CesT_FLAG | Co-expression plasmid for N-terminal His-6 tagged Tir 32-80 and CesT with a C-terminal FLAG tag | This study |
| pCOLADuet-Tir^32-80^-CesT^138^_FLAG | Co-expression plasmid for N-terminal His-6 tagged Tir 32-80 and CesT 2-138 with a C-terminal FLAG tag | This study |
| pCOLADuet- Tir^35-77^-CesT_FLAG | Co-expression plasmid for N-terminal His-6 tagged Tir 35-77 and CesT with a C-terminal FLAG tag | This study |
| pCOLADuet- Tir^35-77^-CesT^138^_FLAG | Co-expression plasmid for N-terminal His-6 tagged Tir 35-77 and CesT 2-138 with a C-terminal FLAG tag | This study |
| pCOLADuet-Tir^32-73^- CesT_FLAG | Co-expression plasmid for N-terminal His-6 tagged Tir 32-73 and CesT with a C-terminal FLAG tag | This study |
| pCOLADuet-Tir^32-73^-CesT^138^_FLAG | Co-expression plasmid for N-terminal His-6 tagged Tir 32-73 and CesT 2-138 with a C-terminal FLAG tag | This study |
| pCOLADuet-Tir^37-80^- CesT_FLAG | Co-expression plasmid for N-terminal His-6 tagged Tir 37-80 and CesT with a C-terminal FLAG tag | This study |
| pCOLADuet-Tir^37-80^-CesT^138^_FLAG | Co-expression plasmid for N-terminal His-6 tagged Tir 37-80 and CesT 2-138 with a C-terminal FLAG tag | This study |
| pCOLADuet-Tir^37-73^- CesT_FLAG | Co-expression plasmid for N-terminal His-6 tagged Tir 37-73 and CesT with a C-terminal FLAG tag | This study |
| pCOLADuet-Tir^37-73^-CesT^138^_FLAG | Co-expression plasmid for N-terminal His-6 tagged Tir 37-73 and CesT 2-138 with a C-terminal FLAG tag | This study |
| pCOLADuet-Tir^490-550^-CesT_FLAG | Co-expression plasmid for N-terminal His-6 tagged Tir 490-550 and CesT with a C-terminal FLAG tag | This study |
| pCOLADuet-Tir^490-550_L514E^-CesT_FLAG | Co-expression plasmid for N-terminal His-6 tagged Tir 490-550 L514E and CesT with a C-terminal FLAG tag | This study |
| pCOLADuet-EspZ- CesT_FLAG | Co-expression plasmid for N-terminal His-6 tagged EspZ and CesT with a C-terminal FLAG tag | This study |
| pCOLADuet-EspZ^L45E^- CesT_FLAG | Co-expression plasmid for N-terminal His-6 tagged EspZ L45E and CesT with a C-terminal FLAG tag | This study |
| pCOLADuet-NleH1- CesT_FLAG | Co-expression plasmid for N-terminal His-6 tagged NleH1 and CesT with a C-terminal FLAG tag | This study |
| pCOLADuet-NleH1^L28E^- CesT_FLAG | Co-expression plasmid for N-terminal His-6 tagged NleH1 L28E and CesT with a C-terminal FLAG tag | This study |
| pCOLADuet-NleH2- CesT_FLAG | Co-expression plasmid for N-terminal His-6 tagged NleH2 and CesT with a C-terminal FLAG tag | This study |
| pCOLADuet-NleH2^L28E^- CesT_FLAG | Co-expression plasmid for N-terminal His-6 tagged NleH2 L28E and CesT with a C-terminal FLAG tag | This study |
| pCOLADuet-NleA- CesT_FLAG | Co-expression plasmid for N-terminal His-6 tagged NleA and CesT with a C-terminal FLAG tag | This study |
| pCOLADuet-NleA^V44E^- CesT_FLAG | Co-expression plasmid for N-terminal His-6 tagged NleA V44E and CesT with a C-terminal FLAG tag | This study |
| pFPV25.1 | *S. enterica* Typhimurium *rpsM* promoter driving gfpmut3 expression | [69] |
| pACYC-GFP | *S. enterica* Typhimurium *rpsM* promoter driving gfpmut3 expression | This study |
| pKNT25-CesT | CesT with C-terminal T25 chimera | This study |
| pKNT25-CesT^138^ | CesT 2-138 with C-terminal T25 chimera | This study |
| pKNT25-Tir^23-550^ | Tir 23-550 with C-terminal T25 chimera | This study |
| pUT18C-CesT | CesT with N-terminal T18 chimera | This study |
| pUT18C- CesT^138^ | CesT 2-138 with N-terminal T18 chimera | This study |
| pUT18C-Tir^23-550^ | Tir 23-550 with N-terminal T18 chimera | This study |
| pUT18C-Tir^81-550^ | Tir 81-550 with N-terminal T18 chimera | This study |
| pUT18C-Tir^23-80^ | Tir 23-80 with N-terminal T18 chimera | This study |
| pUT18C-Tir^490-550^ | Tir 490-550 with N-terminal T18 chimera | This study |
| pUT18C-Tir^23-80_L49E^ | Tir 23-80 L49E with N-terminal T18 chimera | This study |
| pUT18C-Tir^490-550_L514E^ | Tir 490-550 L514E with N-terminal T18 chimera | This study |
| pWSK29-P*_LEE5_* | Complementation vector containing only the *LEE5* promoter | This study |
| pWSK29-P*_LEE5_*-Tir | *tir* complementation vector under the *LEE5* promoter | This study |
| pWSK29-P*_LEE5_*-Tir ^L49E^ | *tir* complementation vector L49E mutant under the *LEE5* promoter | This study |
| pWSK29-P*_LEE5_*-Tir ^L514E^ | *tir* complementation vector L514E mutant under the *LEE5* promoter | This study |
| pWSK29-P*_LEE5_*-Tir ^L49E L514E^ | *tir* complementation vector L49E L514E mutant under the *LEE5* promoter | This study |
| pWSK29-P*_LEE5_*-Tir ^I38E^ | *tir* complementation vector I38E mutant under the *LEE5* promoter | This study |
| pWSK29-P*_LEE5_*-Tir ^500E^ | *tir* complementation vector I500E mutant under the *LEE5* promoter | This study |
| pWSK29-P*_LEE5_*-Tir ^I38E I500E^ | *tir* complementation vector I38E I500E mutant under the *LEE5* promoter | This study |
| pGEN-*luxCDABE* | Lux transcriptional reporter plasmid used for construction of effector plasmids under the em7 promoter | [70] |
| pGEN-MCS | Empty vector control | [70] |
| pGEN-em7-EspZ_FLAG | EspZ_FLAG expression plasmid under control of the em7 promoter | This study |
| pGEN-em7-EspZ^L45E^_FLAG | EspZ_FLAG L45E expression plasmid under control of the em7 promoter | This study |
| pGEN-em7-NleH1_FLAG | NleH1_FLAG expression plasmid under control of the em7 promoter | This study |
| pGEN-em7-NleH1^L28E^_FLAG | NleH1_FLAG L28E expression plasmid under control of the em7 promoter | This study |
| pGEN-em7-NleH2_FLAG | NleH2_FLAG expression plasmid under control of the em7 promoter | This study |
| pGEN-em7-NleH2^L28E^_FLAG | NleH2_FLAG L28E expression plasmid under control of the em7 promoter | This study |
|  |  |  |

**Table B.** **Primers used in this study.**

| Primer | Gene | Direction | Residue | Destination | Sequence |
| --- | --- | --- | --- | --- | --- |
| DL34 | *cesT* | Fwd | 2 | pET28a | GAGCGCTAGCTCAAGATCTGAACTT |
| DL35 | *cesT* | Rev | 138 | pET28a | GAGCCTCGAGCTAATGTTCGTTTTCTAA |
| DL36 | *cesT* | Rev | 156 | pET28a | GAGCCTCGAGTTATCTTCCGGCGTA |
| DL110 | *cesT* | Fwd | H138H | pET28-CesT | GAATAACATTAGAAAACGAACACATGAAAATAGAGG |
| DL111 | *cesT* | Rev | H138H | pET28-CesT | GAGCTAATTTCCTCTATTTTCATGTGTTCGTTTTC |
| DL387 | *cesT* | Rev | 138_FLAG | pCOLADuet-1 | GGAATTCTCGAGTTACTTGTCGTCATCGTCTTTGTAGTCGCCTCCTCTTCCGGCG |
| DL362 | *cesT* | Rev | 156_FLAG | pCOLADuet-1 | GGAATTCTCGAGTTACTTGTCGTCATCGTCTTTGTAGTCGCCTCCTCTTCCGGCG |
| DL142 | *tir* | Fwd | 23 | pCOLADuet-1 | GGAATTGGATCCGTCACAAACAGAC |
| DL143 | *tir* | Fwd | 32 | pCOLADuet-1 | GGAATTGGATCCGGGAGGAACTG |
| DL243 | *tir* | Fwd | 35 | pCOLADuet-1 | GGAATTGGATCCGGGTCATCTAATTAGC |
| DL211 | *tir* | Fwd | 37 | pCOLADuet-1 | GGAATTGGATCCGCTAATTAGCTCTACA |
| DL285 | *tir* | Fwd | 81 | pCOLADuet-1 | GGAATTGGATCCGTCTGAGACATGCTT |
| DL295 | *tir* | Fwd | 490 | pCOLADuet-1 | GGAATTGGATCCGTCAGGGAGCGGC |
| DL212 | *tir* | Rev | 73 | pCOLADuet-1 | GGAATTAAGCTTTCATGGGTTTGTAGGAAG |
| DL244 | *tir* | Rev | 77 | pCOLADuet-1 | GGAATTAAGCTTTCAAGCAAGCCTCGAT |
| DL195 | *tir* | Rev | 80 | pCOLADuet-1 | GGAATTAAGCTTTCATGTAGCTGCAGCAAG |
| DL232 | *tir* | Rev | 550 | pCOLADuet-1 | GGAATTAAGCTTTTAAACGAAACGTACTGG |
| DL288 | *tir* | Fwd | 23 | pUT18C/pKNT25 | GGAATTTCTAGAGCCTTCACAAACAGAC |
| DL289 | *tir* | Rev | 80 | pUT18C/pKNT25 | GGAATTGAGCTCGGTGTAGCTGCAGC |
| DL290 | *tir* | Fwd | 81 | pUT18C/pKNT25 | GGAATTTCTAGAGTCTGAGACATGCTTG |
| DL291 | *tir* | Rev | 550 | pUT18C/pKNT25 | GGAATTGAGCTCGGAACGAAACGTACT |
| DL303 | *tir* | Fwd | 490 | pUT18C/pKNT25 | GGAATTTCTAGAGTCAGGGAGCGGCC |
| DL292 | *cesT* | Fwd | 2 | pUT18C/pKNT25 | GGAATTTCTAGAGTCATCAAGATCTGAACTTTT |
| DL293 | *cesT* | Rev | 138 | pUT18C/pKNT25 | GGAATTGAGCTCGGATGTTCGTTTTCTA |
| DL294 | *cesT* | Rev | 156 | pUT18C/pKNT25 | GGAATTGAGCTCGGTCTTCCGGCGTA |
| DL98 | *LEE5* | Fwd | Promoter | pWSK29 | GGAATTCTCGAGGAAACTTACTGCGCT |
| DL99 | *LEE5* | Rev | Promoter | pWSK29 | GGAATTAAGCTTATCCTTTTATTTAGAAATTTGA |
| DL215 | *tir* | Fwd | 1 | pWSK29-P*_LEE5_* | GGAATTAAGCTTATGCCTATTGGTAACCTTGG |
| DL216 | *tir* | Rev | 550 | pWSK29- P*_LEE5_* | GGAATTGCGGCCGCTTAAACGAAACGTACTG |
| DL311 | *tir* | Fwd | L49E | All | GGATCTCGTTCAGAGTTTTCTCCCCTGAGAAATTC |
| DL452 | *tir* | Rev | L49E | All | TAATGCTCCTGTAGAGCTAATTAGATGACCAGTTCCTCC |
| DL313 | *tir* | Fwd | L514E | All | GTACTTATGCGGAGCTGGCAAACAGCGGCGGATTG |
| DL314 | *tir* | Rev | L514E | All | CTTTGGATACCTTGCCCTGGAGTTCCTATTAACCTTCCGG |
| DL440 | *tir* | Fwd | I38E | All | GGTCATCTAGAGAGCTCTACAGGAGCATTAGGATC |
| DL423 | *tir* | Rev | I38E | All | AGTTCCTCCCCGTGCCGCGCCGTCTG |
| DL424 | *tir* | Fwd | I500E | All | GGAAGGTTAGAAGGAACTCCAGGGCAAGG |
| DL425 | *tir* | Rev | I500E | All | GGTAACTGGGCCGCTCCCTGAAAAATTC |
| DL256 | *tir* | Fwd | 392 | Δ*tir*-NT | GGGGGAATTGGTGCTGGTGTAACGACTGCGCTCCATAGACGAAATCAGGTGTAGGCTGGAGCTGCTTC |
| DL207 | *tir* | Fwd | 50 | Δ*tir*-CT | ACTGGTCATCTAATTAGCTCTACAGGAGCATTAGGATCTCGTTCATTGGTGTAGGCTGGAGCTGCTTC |
| DL208 | *tir* | Rev | 319 | Δ*tir*-CT | CTGCTGTCTGGCCACCTCACCAGCCTCTTTAGCTTGTTGTGCTATTTGCATATGAATATCCTCCTTAG |
| DL223 | *tir* | Fwd | 17 | Δ*tir* | ATGCCTATTGGTAACCTTGGTAATAATGTAAATGGCAATCATTTAATTGTGTAGGCTGGAGCTGCTTC |
| DL224 | *tir* | Rev | 535 | Δ*tir,* Δ*tir*-NT | TTAAACGAAACGTACTGGTCCCGGCGTTGGTGCGGCATTTACAGAACTCATATGAATATCCTCCTTAG |
| DL81 | *escN* | Fwd | 446-Stop | Δ*escN* | TCGGTTCGCTCTGCTTTTACGAATAGATAAAATTCTGTCCAACATACTCAGTGTAGGCTGGAGCTGCTTC |
| DL82 | *escN* | Rev | 9 | Δ*escN* | TCGAACTTAAAGTATTAGGAACGGTAAATGATTTCAGAGCATGATTCTGTCATATGAATATCCTCCTTAG |
| DL257 | *espZ* | Fwd | 2 | pCOLADuet-1 | GGAATTGGATCCGGAAGCAGCAAATTT |
| DL259 | *espZ* | Rev | 98 | pCOLADuet-1 | GGAATTAAGCTTTTAGGCATATTTCATCGC |
| DL457 | *nleH1* | Fwd | 2 | pCOLADuet-1 | GGAATTGGATCCGCTATCACCATCTTCTGT |
| DL458 | *nleH1* | Rev | 293 | pCOLADuet-1 | GGAATTGTCGACCTAAATTTTACTTAATAC |
| DL270 | *nleH2* | Fwd | 2 | pCOLADuet-1 | GGAATTGGATCCGTTATCGCCCTCTTC |
| DL421 | *nleH2* | Rev | 303 | pCOLADuet-1 | GGAATTGTCGACTTATATCTTACTTAATACTACAC |
| DL254 | *nleA* | Fwd | 2 | pCOLADuet-1 | GGAATTGGATCCGAACATTCAACCG |
| DL310 | *nleA* | Rev | 440 | pCOLADuet-1 | GGAATTGCGGCCGCTTAGACTCTTGTTT |
| DL442 | *espZ* | Fwd | 1 | pGEN-em7 | GGAATTTACGTATGGAAGCAGCAAATTTA |
| DL443 | *espZ* | Rev | 98_FLAG | pGEN-em7 | GGAATTGAGCTCTCACTTGTCGTCATCGTCTTTGTAGTCTCCGCCGGCATATTTCATCGC |
| DL453 | *nleH1* | Fwd | 1 | pGEN-em7 | GGAATTTACGTATGCTATCACCATCTTCTGT |
| DL454 | *nleH1* | Rev | 293_FLAG | pGEN-em7 | GGAATTGAGCTCTCACTTGTCGTCATCGTCTTTGTAGTCTCCGCCAATTTTACTTAATAC |
| DL446 | *nleH2* | Fwd | 1 | pGEN-em7 | GGAATTTACGTATGTTATCGCCCTCTTCTA |
| DL447 | *nleH2* | Rev | 303_FLAG | pGEN-em7 | GGAATTGAGCTCTCACTTGTCGTCATCGTCTTTGTAGTCTCCGCCTATCTTACTTAATAC |
| DL448 | *espZ* | Fwd | L45E | pGEN-em7/ pCOLADuet-1 | GTTAGAATCGAAGGTGGAGTTTTAATAGGTGCAGG |
| DL427 | *espZ* | Rev | L45E | pGEN-em7/ pCOLADuet-1 | GCTGCGACTTGTTCCACCTTCTGACTGC |
| DL455 | *nleH1* | Fwd | L28E | pGEN-em7/ pCOLADuet-1 | AGTCGTATTGAATCCTCTGTAAGGGATGCAGC |
| DL456 | *nleH1* | Rev | L28E | pGEN-em7/ pCOLADuet-1 | GTCAGGAGAAGTCAGGTTTCTGGTTAAAGAATTCC |
| DL430 | *nleH2* | Fwd | L28E | pGEN-em7/ pCOLADuet-1 | AATCGTGTTGAATCCTCTGTAAGGGATGCTGC |
| DL431 | *nleH2* | Rev | L28E | pGEN-em7/ pCOLADuet-1 | ATCAGGCGAAGTCAGGTTTCTGGTTAAAGAATTCC |
| DL449 | *nleA* | Fwd | V44E | pCOLADuet-1 | GGATTTGTTGAACATATCCCAGAGGATATGCAG |
| DL429 | *nleA* | Rev | V44E | pCOLADuet-1 | CGTTTCGCATCCATTAGGTAATTCGGATTG |

**S1 Fig. SDS-PAGE analysis of the purified Tir peptide-CesT complexes.** SDS-PAGE analysis of His_6_-Tir peptides co-expressed and purified with (A) CesT and (B) CesT^138^. The resolved samples represent elution fractions from Ni-affinity pull-downs, with Tir residues labeled on top of the corresponding lanes.

**S2 Fig. Gel filtration chromatography of the purified Tir-CesT complexes.** The Tir^23-550^-CesT (black) and Tir^81-550^-CesT (green) complexes elutes at ~180-200 kDa. The Tir^23-80^-CesT (pink), Tir^32-80^-CesT (red), Tir^35-77^-CesT (purple), and Tir^490-550^-CesT (orange) complexes elute at ~50 kDa. CesT (blue) and CesT^138^ (cyan) are shown for reference and elute as dimers at ~36 kDa. Arrows represent molecular weight standards that include ferritin, 440 kDa; conalbumin, 75 kDa; ovalbumin, 44 kDa, ribonuclease A, 13.7 kDa; and aprotinin, 6.5 kDa.

**S3 Fig. Structural comparison of Tir^32-80^-CesT^138^ and EHEC CesT dimers.** Cartoon representation of the (A) Tir^32-80^-CesT^138^ dimer present along the crystallographic 2-fold axis of symmetry, and superposition with (B) EHEC CesT dimer, and (C) crystallographic symmetry mates of EHEC CesT whose domain swapped region superimposes with the Tir binding site. The bottom panel is rotated by 90° outwards. CesT^138^ is coloured purple (β-strands), blue (α-helices), and grey (loops); Tir^32-80^ is coloured pink; EHEC CesT is coloured green with the domain swapped region light green; and the EHEC CesT symmetry mates are coloured orange with the domain swapped region light green.

**S4 Fig. SDS-PAGE analysis of *in vitro* T3SS assays for the various Tir mutagenesis and domain mutants.** EPEC strains grown in T3SS inducing conditions were analyzed for total secreted protein by SDS-PAGE for the (A) Tir β-motif variants, (B) Tir chromosomal truncation mutants, and (C) Tir CesT-extension motif variants. The gels were stained with coomassie brilliant blue G250.
