# Supplementary figures and images for: Molecular basis for CesT recognition of type III secretion effectors in enteropathogenic Escherichia coli
Source: PLoS Pathog. 2018 Aug 17;14(8):e1007224. doi: 10.1371/journal.ppat.1007224 (PMC6114900; doi:10.1371/journal.ppat.1007224)

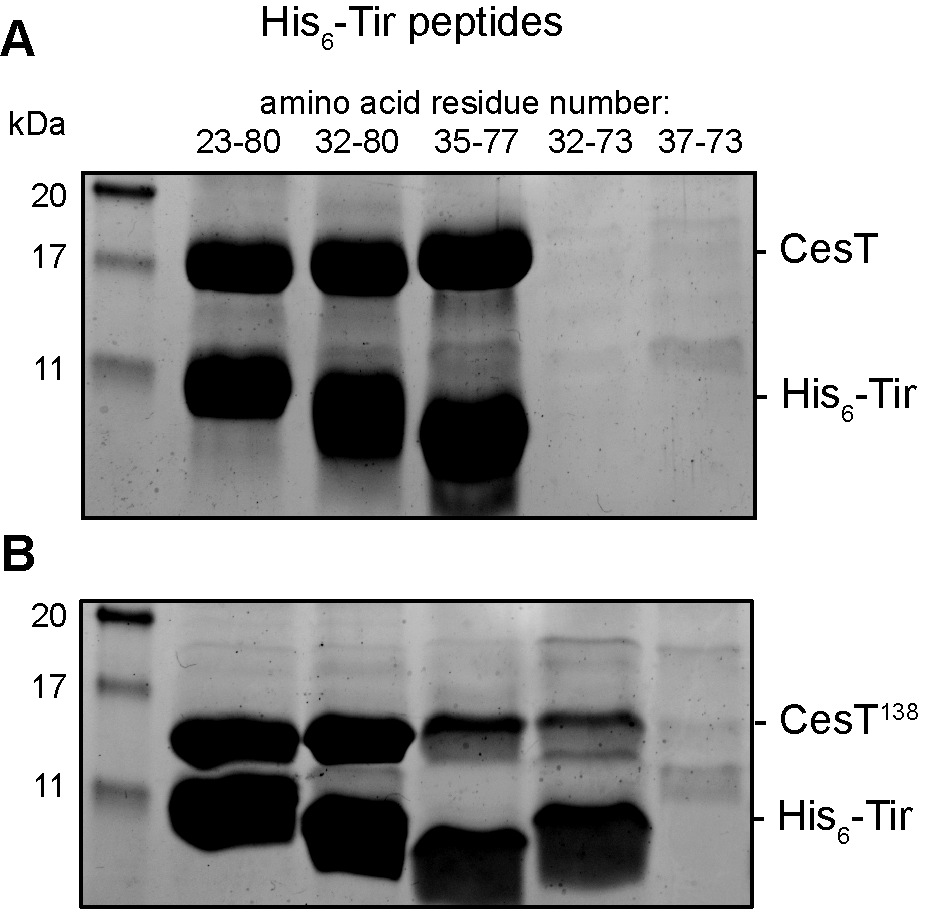

Supplement: S1 Fig — SDS-PAGE analysis of His6-Tir peptides co-expressed and purified with (A) CesT and (B) CesT138. The resolved samples represent elution fractions from Ni-affinity pull-downs, with Tir residues labeled on top of the corresponding lanes. (TIF) [file ppat.1007224.s002.tif]

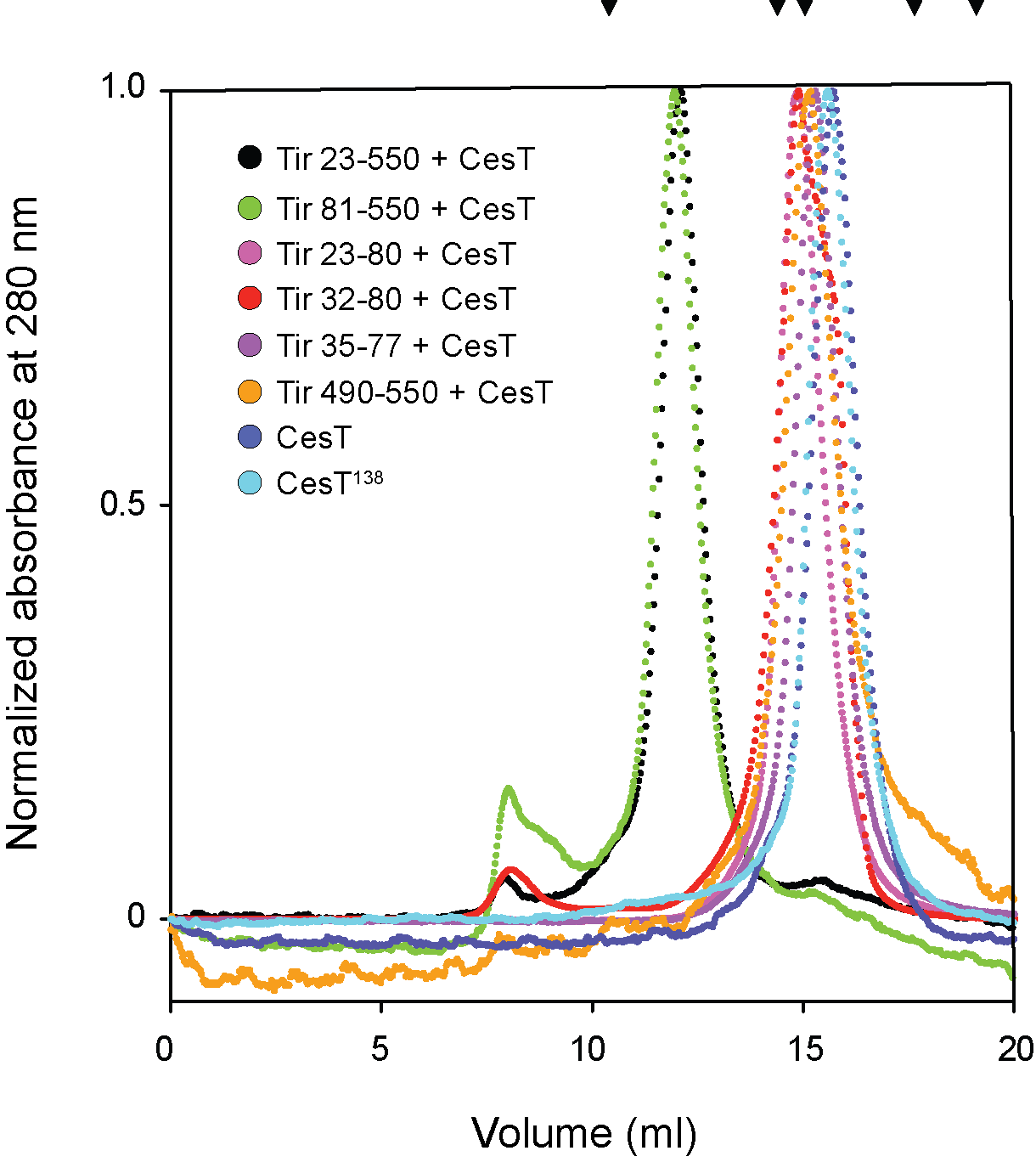

Supplement: S2 Fig — The Tir23-550-CesT (black) and Tir81-550-CesT (green) complexes elutes at ~180–200 kDa. The Tir23-80-CesT (pink), Tir32-80-CesT (red), Tir35-77-CesT (purple), and Tir490-550-CesT (orange) complexes elute at ~50 kDa. CesT (blue) and CesT138 (cyan) are shown for reference and elute as dimers at ~36 kDa. Arrows represent molecular weight standards that include ferritin, 440 kDa; conalbumin, 75 kDa; ovalbumin, 44 kDa, ribonuclease A, 13.7 kDa; and aprotinin, 6.5 kDa. (TIF) [file ppat.1007224.s003.tif]

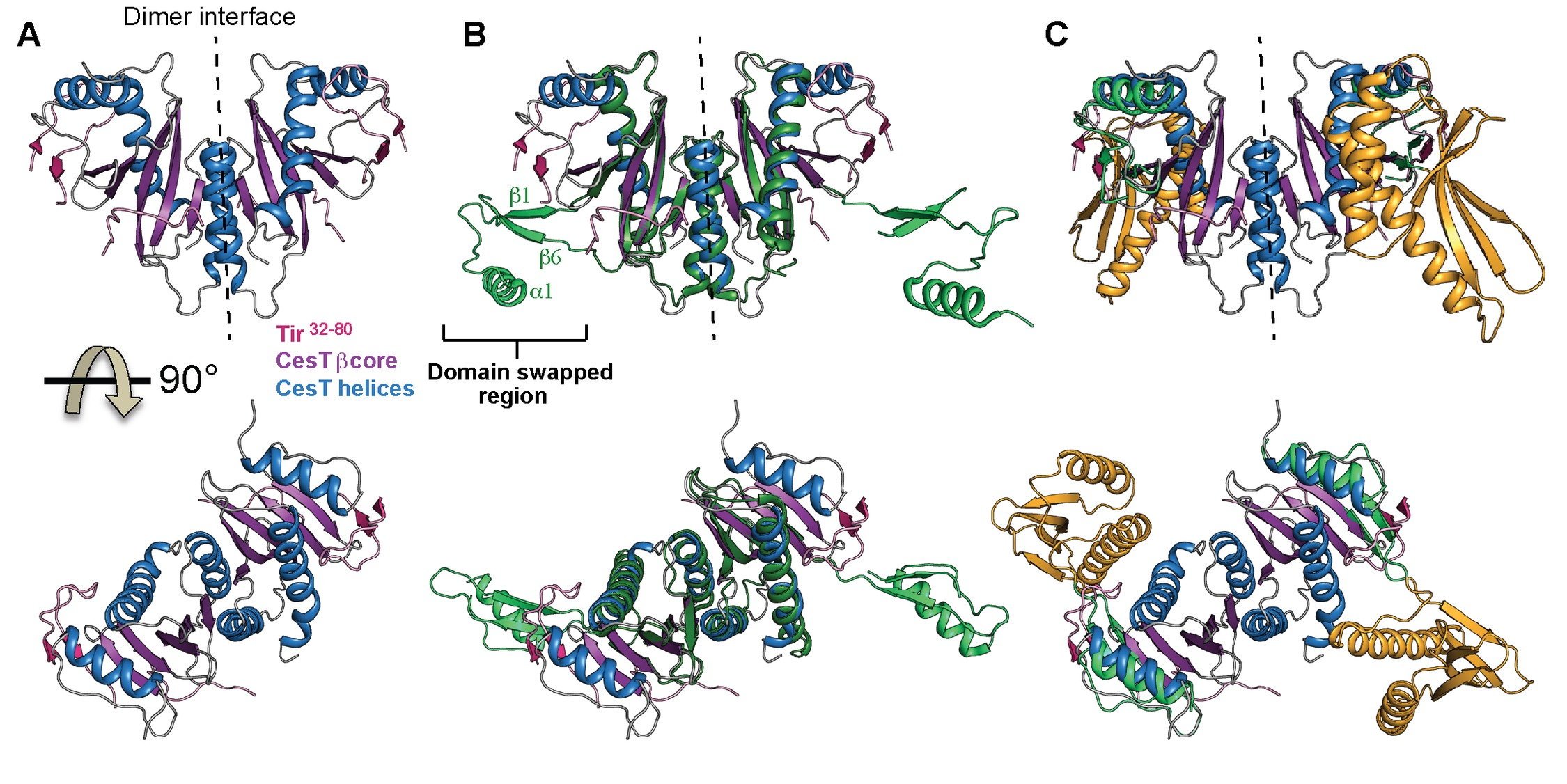

Supplement: S3 Fig — Cartoon representation of the (A) Tir32-80-CesT138 dimer present along the crystallographic 2-fold axis of symmetry, and superposition with (B) EHEC CesT dimer, and (C) crystallographic symmetry mates of EHEC CesT whose domain swapped region superimposes with the Tir binding site. The bottom panel is rotated by 90° outwards. CesT138 is coloured purple (β-strands), blue (α-helices), and grey (loops); Tir32-80 is coloured pink; EHEC CesT is coloured green with the domain swapped region light green; and the EHEC CesT symmetry mates are coloured orange with the domain swapped region light green. (TIF) [file ppat.1007224.s004.tif]

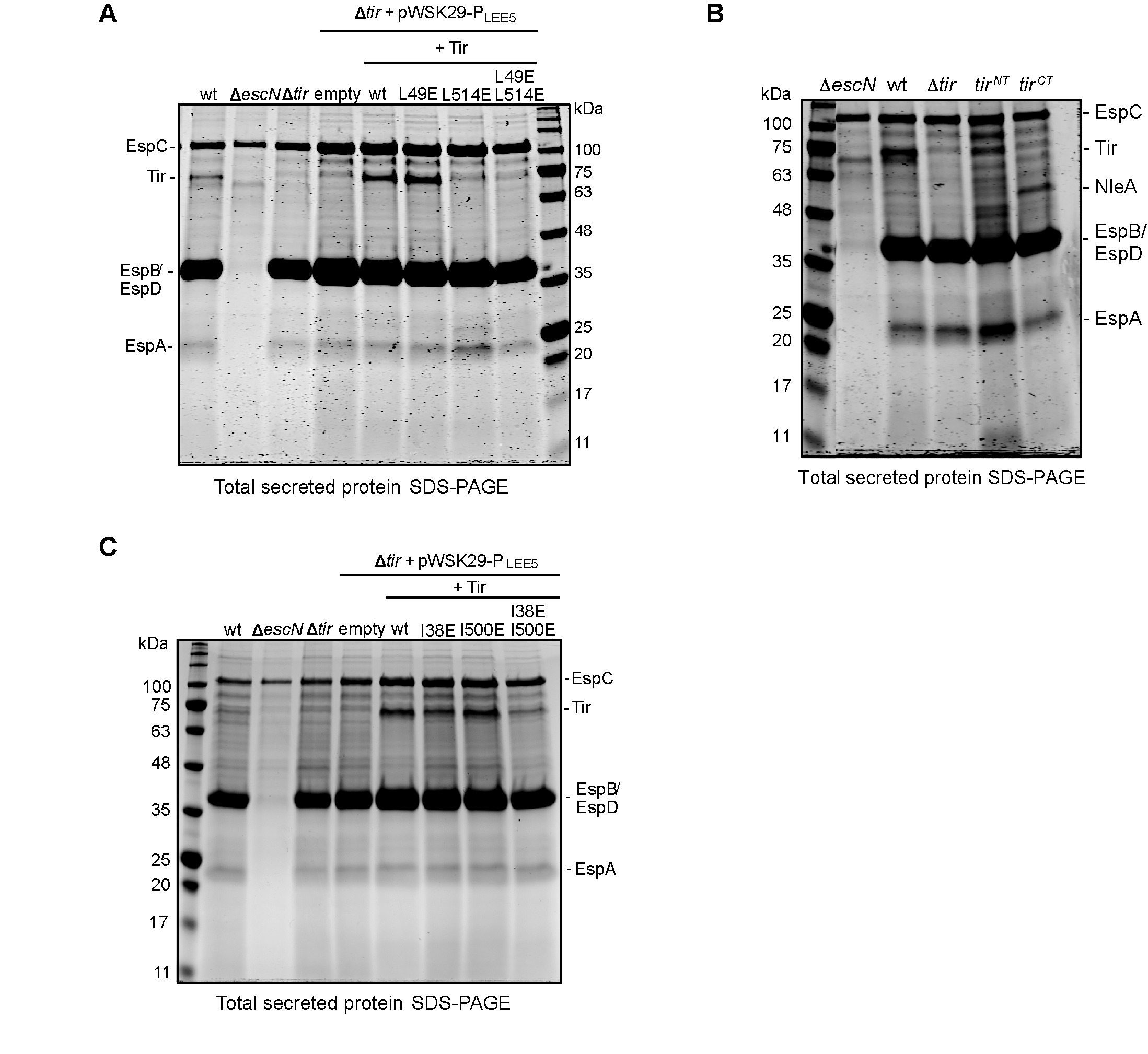

Supplement: S4 Fig — EPEC strains grown in T3SS inducing conditions were analyzed for total secreted protein by SDS-PAGE for the (A) Tir β-motif variants, (B) Tir chromosomal truncation mutants, and (C) Tir CesT-extension motif variants. The gels were stained with coomassie brilliant blue G250. (TIF) [file ppat.1007224.s005.tif]
